# Mechanism of action, potency and efficacy: considerations for cell therapies

**DOI:** 10.1186/s12967-024-05179-7

**Published:** 2024-05-02

**Authors:** Carl G. Simon, Erich H. Bozenhardt, Christina M. Celluzzi, David Dobnik, Melanie L. Grant, Uma Lakshmipathy, Thiana Nebel, Linda Peltier, Anthony Ratcliffe, James L. Sherley, Glyn N. Stacey, Rouzbeh R. Taghizadeh, Eddie H. P. Tan, Sandrine Vessillier

**Affiliations:** 1 Biosystems and Biomaterials Division, National Institute of Standards and Technology (NIST), Gaithersburg, MD USA; 2 United Therapeutics Corporation, Regenerative Medicine Operations, Research Triangle Park, NC USA; 3 Association for the Advancement of Blood and Biotherapies (AABB), Bethesda, MD USA; 4 Niba Labs, Ljubljana, Slovenia; 5 National Institute of Biology, Ljubljana, Slovenia; 6 Department of Pediatrics, Children’s Healthcare of Atlanta, Marcus Center for Cellular and Gene Therapies, Correlative Studies Laboratory, Emory University School of Medicine, Atlanta, GA USA; 7 Pharma Services, Science and Technology, Thermo Fisher Scientific, San Diego, CA USA; 8 Medical Education, Sports Medicine and Orthobiologics, Medical Sales Institute, San Diego, CA USA; 9 Cellular Therapy Lab, Research Institute of McGill University Health Center, Montreal, QC Canada; 10 Synthasome, Inc, San Diego, CA USA; 11 Asymmetrex, LLC, Boston, MA USA; 12 International Stem Cell Banking Initiative, Barley, Herts UK; 13 National Stem Cell Resource Centre, Institute of Zoology, Chinese Academy of Sciences, Beijing, China; 14 Beijing Institute for Stem Cells and Regenerative Medicine, Chinese Academy of Sciences, Beijing, China; 15 Kendall Innovations, Cambridge, MA USA; 16 Cell and Gene Therapy Facility, Health Sciences Authority, Singapore, Singapore; 17 Science, Research and Innovation Group, Biotherapeutics and Advanced Therapies Division, Medicines and Healthcare Products Regulatory Agency, South Mimms, Hertfordshire UK

**Keywords:** Cell therapy product, Efficacy, Efficacy endpoint, Efficacy endpoint test, Mechanism of action, Potency, Potency test

## Abstract

One of the most challenging aspects of developing advanced cell therapy products (CTPs) is defining the mechanism of action (MOA), potency and efficacy of the product. This perspective examines these concepts and presents helpful ways to think about them through the lens of metrology. A logical framework for thinking about MOA, potency and efficacy is presented that is consistent with the existing regulatory guidelines, but also accommodates what has been learned from the 27 US FDA-approved CTPs. Available information regarding MOA, potency and efficacy for the 27 FDA-approved CTPs is reviewed to provide background and perspective. Potency process and efficacy process charts are introduced to clarify and illustrate the relationships between six key concepts: MOA, potency, potency test, efficacy, efficacy endpoint and efficacy endpoint test. Careful consideration of the meaning of these terms makes it easier to discuss the challenges of correlating potency test results with clinical outcomes and to understand how the relationships between the concepts can be misunderstood during development and clinical trials. Examples of how a product can be “potent but not efficacious” or “not potent but efficacious” are presented. Two example applications of the framework compare how MOA is assessed in cell cultures, animal models and human clinical trials and reveals the challenge of establishing MOA in humans. Lastly, important considerations for the development of potency tests for a CTP are discussed. These perspectives can help product developers set appropriate expectations for understanding a product’s MOA and potency, avoid unrealistic assumptions and improve communication among team members during the development of CTPs.

**Supplementary Information:**

The online version contains supplementary material available at 10.1186/s12967-024-05179-7.

## Introduction

### Challenges of MOA and potency

Determining the MOA and developing adequate potency tests remain challenging for CTPs [1–6]. US regulations require that CTPs have a potency test for licensure. Potency tests should be based on the product’s MOA [7, 8]. The goal of a potency test is to assure that the product is able to achieve its intended mechanism of action. Other major roles of potency tests are to assess manufacturing consistency and product stability. Potency tests are often bioassays that involve measuring a response in cells.

For many of the 27 US FDA-approved CTPs (as of February 2024), the relationships between the potency tests and proposed MOAs are unclear. Table 1 highlights representative examples of the information regarding potency and MOA taken from the regulatory documentation for seven of the FDA-approved CTPs (see Supplementary File 1 for information about all 27 CTPs). The FDA has a useful website containing information for approved CTPs [9]. Two documents posted for each product are particularly useful: the “Product Insert” and “Summary Basis for Regulatory Action” (SBRA), which contain information about the clinical trials, product testing, potency and MOA. The regulatory documentation for Provenge, Gintuit, MACI and Amtagvi indicate that the MOA is not known for these products (Table 1). For Kymriah, the documentation states that it is difficult to correlate the potency test results with efficacy. The documentation for Stratagraft discusses data regarding the product activity but does not directly state whether this activity is to be regarded as the MOA. For Rethymic and Lantidra, the documentation uses the words “proposed” and “believed” when discussing the MOA to indicate uncertainty. The lack of clarity regarding potency and MOA lead to the question: “Why is there such a challenge with MOA and potency?” This perspective article attempts to shed light on this question.

**Table 1:** Regulatory comments regarding potency & MOA For FDA-approved cell therapies

Product (year approved, company): Description	Indication	Potency test	MOA
Provenge (2011, Dendreon): Autologous cellular immunotherapy (CD54 + cells activated with PAP-GM-CSF)	Prostate cancer	Surface expression of CD54 (ICAM-1) on APCs after culture with PAP-GM-CSF (flow cytometry)	*Package Insert:* “While the precise **mechanism of action is unknown**, PROVENGE is designed to induce an immune response targeted against PAP, an antigen expressed in most prostate cancers.”
Gintuit (2012, Organogenesis): Allogeneic cultured keratinocytes and fibroblasts in bovine collagen	Oral mucogingival defects	Histology with morphological assessments	*Package Insert:* “GINTUIT does not function as a tissue graft. The MOA by which GINTUIT increases keratinized tissue at the treated site has not been identified.” *FDA SBRA:* “The histology assay is a good measure of the structural integrity of the product, however**, the assay is not an adequate, sensitive measure of biological activity**.”
MACI (2016, Vericel): Autologous cultured chondrocytes on porcine collagen membrane	Knee cartilage defects	PCR measurement of aggrecan gene expression	*Package Insert:* “No clinical pharmacology studies have been conducted with MACI and a **mechanism of action has not been established**.”
Kymriah (2017, Novartis): CD19-directed genetically modified autologous T-cell immunotherapy	B-cell acute lymphoblastic leukemia	Interferon-γ production by test article upon stimulation with CD19 + cells	*Package Insert:* “Upon binding to CD19-expressing cells, the CAR transmits a signal to promote T-cell expansion, activation, target cell elimination, and persistence of the KYMRIAH cells” *FDA Briefing Document:* “In the clinical trials, IFN-γ production varied greatly from lot-to-lot, **making it difficult** to correlate IFN-γ production with in vitro safety or efficacy.”
Stratagraft (2021, Stratatech): Allogeneic cultured keratinocytes and dermal fibroblasts in murine collagen	Thermal burns	*Not reported*	*Package Insert:* “In vitro studies have shown that Stratagraft **secretes** human growth factors and cytokines and contains human ECM proteins.”
Rethymic (2021, Enzyvant): Allogeneic processed thymus tissue	Athymia	Histology	*Package Insert:* “The **proposed** mechanism of action involves the migration of recipient T cell progenitors from the bone marrow to the implanted Rethymic slices, where they develop into naïve immunocompetent recipient T cells.”
Lantidra (2023, CellTrans): Allogeneic pancreatic islets	Type 1 diabetes	i) insulin release in glucose stimulated islets; ii) islet yield by microscopy; iii) viability by microscopy	*Package Insert:* “The primary mechanism of action of LANTIDRA is **believed** to be secretion of insulin by infused (transplanted) β- cells.”
Amtagvi (2024, Iovance): Autolologous T-cell therapy made of ex vivo-expanded lymphocytes harvested from patient tumors	Melanoma	i) redacted, ii) redacted, iii) redacted, iv) redacted, v) dose (total viable cells), vi) redacted and vii) redacted	*Package Insert:* ““The specific mechanism of action of AMTAGVI (lifileucel) is **unknown**.”

*FDA* Food and Drug Administration, *PAP-GM-CSF* human prostatic acid phosphatase (PAP), an antigen expressed in prostate cancer tissue, linked to human granulocyte–macrophage colony-stimulating factor (GM-CSF), an immune cell activator, *SBRA* Summary Basis for Regulatory Action. Bolded text highlights areas of uncertainty or concern and bolding was added by the authors

*Source:* https://www.fda.gov/vaccines-blood-biologics/cellular-gene-therapy-products/approved-cellular-and-gene-therapy-products

Table 2 summarizes information on potency tests for the 27 approved CTPs. Supplementary File 1 summarizes the key aspects of each of the FDA-approved CTPs including product name, year approved, sponsor, product description, indication, clinical trial structure, efficacy endpoints, MOA, potency test, comments and references. This information was used as background for many of the points highlighted in this perspective.

**Table 2:**
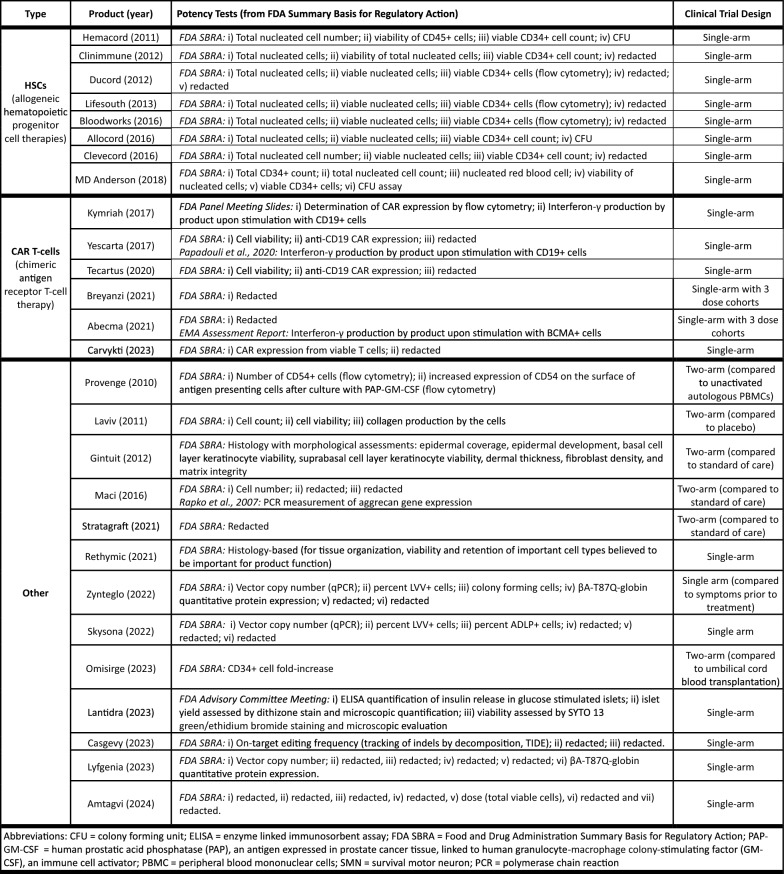
Potency tests and clinical trial designs for the 27 US-approved therapies containing live human cells

### Correlation of potency test with clinical outcome

It is desirable for the potency test to reflect clinical efficacy [7, 8]. Manufactured units that show high potency via the potency test should tend to show an efficacious benefit to patients, while units with lower potency, as measured by the potency test, should have a less efficacious benefit to patients. However, a correlation between the potency test and clinical efficacy is not required. If a product is efficacious and the risk–benefit profile is acceptable, then a product may receive regulatory marketing clearance even if the potency test does not correlate with efficacy endpoint test results (Table 1). For CTPs, MOAs may not be fully understood making it difficult to relate MOAs to potency or efficacy.

### Kymriah: relationship between potency test results and clinical outcome

**Fig. 1:**
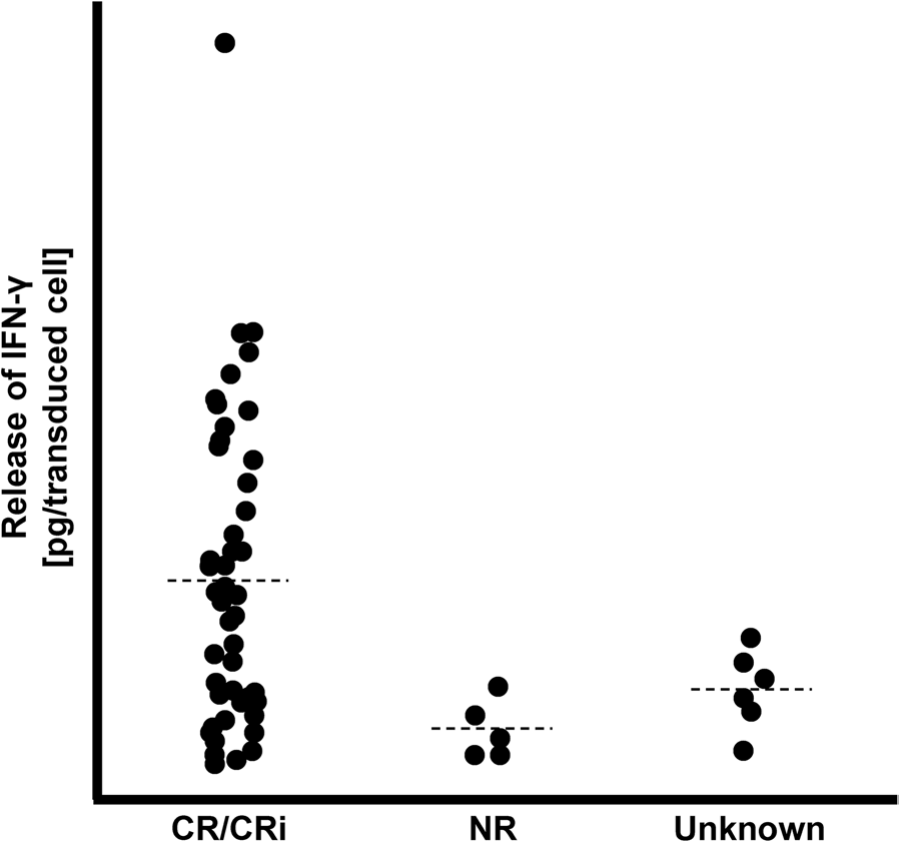
Relationship between potency test results and efficacy endpoint for Kymriah clinical trial for the treatment of pediatric acute lymphoblastic leukemia after three months. The potency test was interferon-γ (IFN) production by test article upon stimulation with CD19 + cells. Data are adapted from FDA Advisory Committee Meeting materials [10]. Data are from 63 patients: 52 CR/CRi, 5 NR and 6 Unknown. CR = complete remission; CRi = complete remission with incomplete blood count recovery; NR = nonresponder; Unknown = unknown response

In 2017, Kymriah (tisagenlecleucel) was the first chimeric antigen receptor (CAR) T-cell therapy that was cleared for marketing by FDA. Kymriah is used to treat leukemia and potency was defined as the ability of the CAR T-cells to secrete interferon-γ (IFN-γ) following exposure to target cells expressing CD19. The FDA held a meeting of the Oncologic Drugs Advisory Committee to discuss this revolutionary technology in a public forum. The online documentation contained a noteworthy graph showing the relationship between potency measurements and efficacy endpoints (Fig. 1) [10]. To the authors’ knowledge, this is the only publicly available data to show the relationship between potency testing results and clinical outcome for a US-approved CTP. The graph shows that the potency test results correlated with remission, but that there was overlap between responders and non-responders. The FDA Briefing Document from the meeting stated “In the clinical trials, IFN-γ production varied greatly from lot-to-lot, making it difficult to correlate IFN-γ production in vitro to tisagenlecleucel safety or efficacy” (Table 1) [11].

## Definitions

**Fig. 2:**
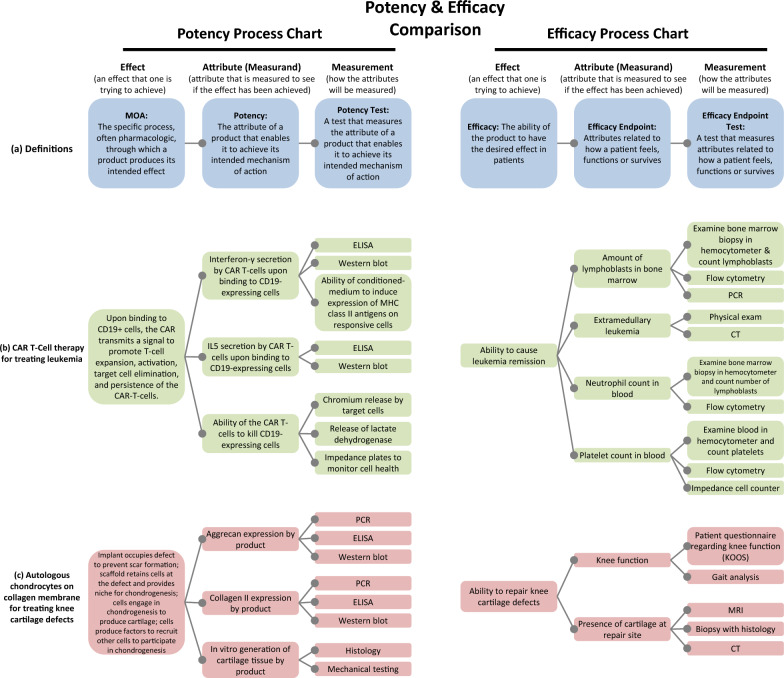
Potency and efficacy process charts. Comparison of potency and efficacy definitions and processes for two example products demonstrates the parallelism between the two processes. **a** Definitions of the components of a potency and efficacy process chart. **b** Potency and efficacy process charts for treating leukemia with a CAR T-cell therapy (based on Kymriah). **c** Potency and efficacy process charts for a product for treating knee cartilage defects with chondrocytes on a collagen membrane (based on MACI). The figure graphically represents the relationships among the three inherent components of any analytical assessment: effect; attribute (measurands); and measurement; for both potency and efficacy. The examples are meant to illustrate concepts and are not intended as recommendations for potency tests or efficacy endpoint tests. The charts are not comprehensive and there may be other MOAs, attributes or tests that are not mentioned. *CT* computed tomography scan, *ELISA* enzyme linked immunosorbent assay, *IL5* interleukin 5, *KOOS* knee injury and osteoarthritis outcome score, *MHC* major histocompatibility, *MRI* magnetic resonance imaging, *PCR* polymerase chain reaction. The cited sources were used to assemble the content for (**b**) Kymriah [10, 11, 24–32] and (**c**) MACI [33–37]

Clear definitions are critical to improving the understanding of the relationships among MOA, potency and efficacy. Figure 2a provides definitions of 6 key terms and delineates their relationships as discussed below. Definitions for terms were adapted from existing definitions but may not be identical to definitions in the source documents. Definitions were created for terms that had not previously been defined.

### Measurand and measurement

Since the current discussion is focused on a product attribute (potency) and how to measure it (potency test), it is important to understand the key metrological term, measurand.

Measurand: “*the quantity or property intended to be measured*” [12]

A measurand is the attribute or property of a material that is being assessed. A measurand and a material attribute can be the same thing when the goal of a measurement is to measure the material attribute. The use of the word “intended” in this definition is intentional and important. This is because it may be impossible to measure what one intends to measure. Further, due to experimental artifacts, what was measured during the measurement may not be what was intended [13]. These points are reminders that there are no perfect measurements and that all measurements have false positives and false negatives. This concept is key to misunderstandings surrounding potency tests.

It is important to independently define the “measurand” (the attribute being measured) and the “measurement”, according to the International Vocabulary of Metrology (VIM) [12].

Measurement: *“process of experimentally obtaining one or more quantity values that can reasonably be attributed to a quantity”* [12]

There may be a variety of different methods for making a measurement of a material attribute and the results of the measurements may not agree. For example, two labs may try to make the same measurement but get different results. It may be “that different experiments are inadvertently being performed (*i.e.*, there are critical experimental differences that are not accounted for)” [14]. This is not a new challenge for cellular therapies but has existed more broadly for biomedical research.

### Mechanism of action (MOA), potency, potency test, efficacy, efficacy endpoint and efficacy endpoint test

Following are six key definitions that are charted in Fig. 2a. These definitions have been adapted from the cited sources. The source definitions are provided in Supplementary File 2 as a reference.

- *Mechanism of action (MOA):* The specific process, often pharmacologic, through which a product produces its intended effect [15, 16]
- *Potency:* The attribute of a product that enables it to achieve its intended mechanism of action [17–19]
- *Potency Test:* A test that measures the attribute of a product that enables it to achieve its intended mechanism of action [20]
- *Efficacy:* The ability of the product to have the desired effect in patients [21]
- *Efficacy Endpoint:* Attributes related to how a patient feels, functions or survives [22, 23]
- *Efficacy Endpoint Test:* A test that measures attributes related to how a patient feels, functions or survives (the authors are not aware of any existing definition for this term)

It is key that these six concepts be defined separately from one another in order to understand how they relate to one another.

### Potency ≠ efficacy

A common mistake is to assume that potency and efficacy are the same thing and that a potency test that measures potency is also a measure of efficacy. This cannot be true since efficacy can only be measured by clinical response. Potency tests are laboratory assays which may or may not be a predictor of clinical response. Another way to think about it is “potency is laboratory” whereas “efficacy is clinical”; and the two are tied together by the MOA.

## Potency process charts and efficacy process charts

The definitions given in Fig. 2a are applied to practical examples of two approved CTPs: a CAR T-cell therapy in Fig. 2b [10, 11, 24–32] (based on Kymriah) and tissue-engineered chondrocytes on a collagen membrane in Fig. 2c [33–37] (based on MACI). Each chart is composed of 2 triads: an effect, an attribute and a measurement which are applied to MOA in the first three boxes and to efficacy in the fourth, fifth and sixth boxes. Another way to think of these triads is 1) an effect that one is trying to achieve, 2) an attribute that can be measured to see if the effect has been achieved (the measurand), and 3) how the attributes will be measured (the measurement).

The goal of the potency and efficacy process charts in Fig. 2a is to distinguish and independently define each of the 6 components, making it easier to understand how their interrelationships can break down during product development and clinical trials.

### Separate MOA from potency

A benefit of separating the “MOA” from the “potency” attribute is that it allows for the defined potency attribute to be incorrect. For example, potency of a CAR T-cell therapy could be defined as its ability to secrete IFN-γ upon recognizing target cells (Fig. 2b). However, it could be that IFN-γ secretion is not involved in the proposed MOA of the product (target cell elimination) and the product may have an unknown MOA.

Separation of “MOA” from the “potency” attribute also allows different biological activities related to the MOA to be defined as potency. For example, the potency of a CAR T-cell therapy could be defined as its ability to secrete IFN-γ upon binding to target cells (Fig. 2b), its ability to secrete IL5 upon binding to target cells or the ability of the CAR T-cells to kill target cells [31]. All, some or none of these could be solely indicative of the MOA. It may be that IFN-γ secretion is an essential factor in the MOA, and that IL5 and cell killing are not major contributors. Or, maybe none of these activities are part of the MOA.

### Separate potency from potency test

Likewise, “potency” and “potency test” should be distinct in the event that 1) the “potency test” is not a reliable measurement of the potency attribute and 2) there are multiple ways to measure the potency attribute. For case 1), measurement of IFN-γ secretion by CAR T-cells by enzyme linked immunosorbent assay (ELISA) (Fig. 2b) could lead to artifactual data that do not accurately measure the amount of IFN-γ released. For case 2, IFN-γ release could be measured by ELISA or Western blot and the results may not agree. These possibilities are obscured if the potency attribute is defined in terms of a specific measurement, emphasizing the value in keeping potency (attribute or measurand) and the potency test (measurement) distinct.

### Separate efficacy from efficacy endpoint

The concept of “efficacy” should remain generalized and distinct from the “efficacy endpoint”, while the “efficacy endpoint” should be specific to a patient symptom or attribute. For instance, when developing a CAR T-cell therapy, developers could define “efficacy” as “ability to cause leukemia remission”, which is a general definition that does not mention patient attributes or symptoms (i.e., the “efficacy endpoints”) (Fig. 2b). If efficacy is defined as “amount of lymphoblasts” (instead of as the “ability to cause leukemia remission”), then developers are led to believe that “amount of lymphoblasts” is certain to be indicative of the disease status of a patient, and the possibility that “amount of lymphoblasts” is not linked to the disease state is more easily overlooked. The measurement of the “amount of lymphoblasts” in bone marrow may not be indicative of a particular blood cancer, and it may be that other efficacy endpoints should be considered, such as presence of extramedullary tumors. Having separate definitions for “efficacy” (the effect you are trying to achieve) and “efficacy endpoints” (patient attributes or symptoms) allows for the possibility that a given “efficacy endpoint” is not indicative of the disease state. This distinction is subtle but it avoids the false assumptions that lead to confusion when unexpected clinical results are observed.

### Separate “efficacy endpoint” from “efficacy endpoint test”

Separation of the “efficacy endpoint” from the “efficacy endpoint test” (the measurement) makes it easier to deal with false measurement results. The VIM provides separate definitions for measurand and measurement [12], so it makes sense to do the same here. For example, the amount of lymphoblasts in bone marrow may be used as an efficacy endpoint for leukemia treatment (Fig. 2b). The measurement of the amount of lymphoblasts could be conducted by hemocytometer counting (efficacy endpoint test) but the results could be deceptive. There may be an interference in the hemocytometer count (e.g., cell clumping, debris) causing it to give false readings. The efficacy endpoint should be defined without referring to a specific measurement method, so that the possibility of false results is not obscured.

Separation of the “efficacy endpoint” from “efficacy endpoint test” makes it easier to deal with different measurements of an attribute. The number of lymphoblasts in marrow could be measured by counting in a hemocytometer, by PCR (polymerase chain reaction) or by flow cytometry; and the three measurements may not agree [30]. If the efficacy endpoint is defined as a hemocytometer measurement of lymphoblasts, then the circumstance of multiple measurements that may not agree becomes intractable.

## “Potent but not efficacious” and “not potent but efficacious”

Since potency and efficacy cannot be the same thing (because potency is measured by a lab test and efficacy is measured by clinical trial), then it follows that a therapeutic could be “potent but not efficacious” or “not potent but efficacious.” These are confusing scenarios, but it is important to consider them, since they get to the heart of the challenge surrounding MOA, potency and efficacy.

### Potent but not efficacious: wrong patient population

An example of “potent but not efficacious” is giving a potent chemotherapeutic to a patient with bronchitis. Imagine that the product is truly potent for its intended MOA and is truly efficacious when used as intended in the treatment of cancer. Yet, the chemotherapeutic will not help the patient with bronchitis. This could be a case of a misdiagnosis or treating the wrong patient population.

This example could seem irrelevant, but identifying the appropriate indication and selecting the appropriate patient inclusion and exclusion criteria for a clinical trial are critical. When statistical significance is not observed in a costly and lengthy clinical trial, there are often controversial post-hoc subgroup analyses that identify responsive cohorts. A better understanding of MOA helps with identification of patients most likely to respond prior to starting a clinical trial. An example is companion diagnostics [38], such as the well-known example of breast cancer screening to treating HER2/*neu*-positive patients with Herceptin, an anti-HER2/*neu* antibody drug [39]. The FDA website currently lists 169 companion diagnostics, which highlights the value of identifying the correct patient population [38].

Another example is the indication for Kymriah, which is carefully worded with 5 qualifiers to specifically identify those patients most likely to benefit from treatment: “patients up to 25 years of age with B-cell precursor acute lymphoblastic leukemia (ALL) that is refractory or in second or later relapse” [28] (Additional file 1). Another example is the carefully worded indication for Lantidra which has six qualifiers: “The treatment of adults with Type 1 diabetes who are unable to approach target HbA1c because of current repeated episodes of severe hypoglycemia despite intensive diabetes management and education.” Careful thinking and strategy are required to write CTP indications so that patients for whom the product is most likely to be efficacious are identified.

### Potent but not efficacious: incorrect hypothesis regarding the disease mechanism

Another example of “potent but not efficacious” is having an incorrect hypothesis regarding the MOA for the targeted indication. Imagine the therapeutic is truly potent and achieves its intended MOA. Yet, it does not benefit the patient population because the hypothesis regarding the MOA is wrong for the intended indication. Achieving the intended MOA does not have an effect in treating the intended indication. This exercise highlights the importance of separating the concepts of potency and efficacy. If potency and efficacy are equated to one another, then “potent but not efficacious” becomes inconceivable.

Another useful example for understanding “potent but not efficacious” is as follows. If a person takes an aspirin for a headache and the headache is not alleviated, does that mean the aspirin is not potent? The aspirin is probably still potent, inhibits cyclooxygenase and blocks prostaglandin synthesis to achieve its intended MOA [40]. Yet, the aspirin is not efficacious in curing the headache. The headache may be caused by factors unrelated to cyclooxygenase or prostaglandins.

### Not potent but efficacious: alternate MOA

An example of “not potent but efficacious” could be a therapeutic that is effective due to an MOA that is not the proposed MOA. Imagine the therapeutic is truly *not potent* for its intended MOA, but the therapeutic is truly effective in treating the intended indication. It could be that the proposed MOA was incorrect, and the product is effective due to an alternate MOA (perhaps an unknown biological activity). To revisit the CAR T-cell example discussed in Fig. 2b, potency of a CAR T-cell therapy may not be due to its ability to secrete IFN-γ upon binding to target cells, but instead could be due to secretion of IL5 upon binding to target cells or the ability of the CAR T-cells to kill target cells via perforin-granzyme or Fas–Fas ligand interactions [41].

### Not potent but efficacious: false negative potency test

Another example of “not potent but efficacious” is a false negative potency test result. Imagine that a therapeutic is truly potent. It achieves its intended MOA and achievement of the intended MOA is truly effective in treating the intended indication. Yet, the potency test indicates that the therapeutic is not potent. In this scenario, the potency test may be a poor measure of true potency and may yield incorrect results: that the therapeutic is not potent. In this case, the potency test is giving false negative results. For example, the potency test may not be stable and reagent degradation results in intermittent false negatives.

## MOA case studies: aspirin and acetaminophen

### Aspirin MOA

The MOA of aspirin (acetylsalicylic acid) is useful to consider. Aspirin is considered the most widely used drug of all time [42]. For thousands of years, plant extracts, such as from willow bark which contains salicylate, have been used to treat rheumatism (joint inflammation) and pain. In 1897, Bayer reported a synthetic version of acetylsalicylic acid that was named aspirin. In the early 1970s, research by John Vane shed light on aspirin’s MOA with the discovery of cyclooxygenase (COX-1) and its acetylation by aspirin [43–45], leading to the 1982 Nobel Prize in Medicine [46]. However, other COX isoforms were discovered; COX-2 in 1991 [47] and COX-3 in 2002 [48]; and COX-3 was inhibited by aspirin. In the 2000s, additional MOAs for aspirin were elucidated: i) uncoupling of oxidative phosphorylation [49], ii) promotion of nitric oxide synthesis to inhibit inflammation [50] and iii) inhibition of NF-κB (nuclear factor kappa-light-chain-enhancer of activated B cells) activation to inhibit inflammation [51]. This evidence suggests that aspirin has multiple (and possibly multifactorial) MOAs and can affect at least 5 pathways: COX-1, COX-3, oxidative phosphorylation, nitric oxide and NF-κB. Future studies may identify new MOAs for aspirin. Thus, even after thousands of years of use, and being the most widely used drug of all time, the MOA of aspirin is still unclear.

### Acetaminophen MOA

Another example of a widely used drug whose MOA is uncertain is acetaminophen (paracetamol). Acetaminophen, a small molecule drug with a molecular mass of 151 g/mol, is probably the most widely prescribed drug for children and is used for pain management and fever reduction. It was first synthesized in 1878, first used clinically in 1887, and was first marketed in the US in the 1950s [52]. Acetaminophen’s package insert states that “The precise mechanism of the analgesic and antipyretic properties of acetaminophen is not established but is thought to primarily involve central actions” [53]. There is evidence that acetaminophen inhibits prostaglandin synthesis [54], inhibits COX-3 [48], activates a vanilloid receptor (TRPV1) [55] and modulates the cannabinoid system [56].

### CTPs vs. “aspirin and acetaminophen”

Aspirin and acetaminophen, small molecule drugs with molecular masses of 180 g/mol and 151 g/mol, respectively, each has multiple activities. In small molecule drug discovery, the term polypharmacology means “the binding of a drug to multiple target proteins, with clinical effects being mediated through the modulation of the set of protein targets” [57]. The precise MOA for many drugs is unknown, which indicates that knowing the MOA is desirable but unnecessary for having an effective therapeutic [58]. A cell therapy delivers millions of metabolically active cells to a patient, where each cell is a bag of thousands of different molecules, including proteins, lipids, carbohydrates and nucleic acids, and each of these molecules could have multiple activities. Given the fact that the MOAs for aspirin and acetaminophen are still being investigated, determining the precise MOA for a cell-containing therapeutic seems especially challenging. Further, without a good understanding of the MOA, it is hard to imagine that there will be good potency tests, given that potency tests should be based on the MOA. Given the tools currently at our disposal, it may be useful to consider the establishment of a product’s MOA as an aspirational goal, instead of as something that can be known with certainty. This may change with development of improved technology for determining MOA.

## Clinical trials are not designed to establish MOA

### Example application: establishing MOA for a CAR T-cell product

It is useful to consider an example application for establishing the MOA of a CAR T-cell therapy directed against leukemia cells expressing a target surface antigen (based on Kymriah). The proposed MOA for this product is that upon binding to CD19 + cells (cancer cells), the CAR transmits a signal to promote T-cell expansion, activation, target cell elimination, and persistence of the CAR-T-cells.

Table 3 shows experiments that can be used to establish the MOA. The tests can be run in cell culture using an in vitro cell killing assay, in animals using an animal model for leukemia and in a human clinical trial to treat leukemia patients. The test article is “CAR T-cells directed against leukemia cells expressing a target surface antigen” as shown in the first row of the table. The second row is a negative control, to assess if the test article has an effect above background signal. The negative control could be an untreated well for cell culture, could be a sham CAR T-cell delivery for the animal (saline injection) and may not be possible for humans since it may not be ethical to leave humans untreated. The third row is “Standard of care,” which is not applicable for cell culture, but could be an existing chemotherapy for animals and humans. The fourth row is “Unmodified T-cells” only, which can establish if CAR expression is required for the MOA. The fifth row is “CAR T-cells with empty CAR,” which can establish if a specific CAR must be expressed or if any CAR can suffice. The sixth row is “Dead CAR T-cells” which tests if live cells are required. Finally, the seventh row is “Fibroblasts expressing CAR” to determine if T-cells are required or if any cell can suffice. Note that most experiments in Table 3 can be done in cells or animal models, but, due to ethical considerations, only one or two of the experiments can be done in CTP clinical trials. This example application highlights the difficulty in establishing MOA in humans. Clinical trials are designed to assess efficacy and safety, not to establish MOA or validate a potency test.

**Table 3:**
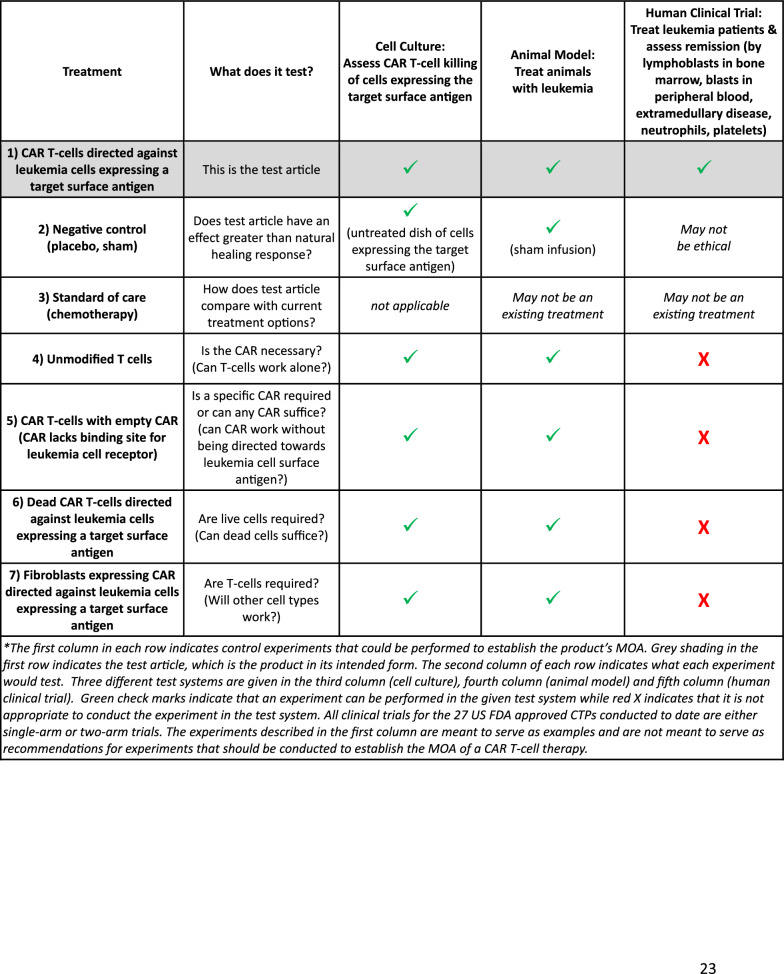
Example application for establishing MOA for a hypothetical product: chimeric antigen receptor (CAR) T-cell therapy directed against leukemia cells expressing a target surface antigen (based on Kymriah)

### Example application: establishing MOA for a tissue engineered medical product

Another example application is given in Table 4: establishing the MOA of a tissue engineered medical product composed of autologous cultured chondrocytes on a collagen membrane for treating knee cartilage defects (based on MACI). The proposed MOA is cartilage regeneration by the following activities: i) the implant will occupy the trauma site to prevent scar formation, ii) the cells will proliferate and secrete cartilage matrix that will form new cartilage tissue, iii) the scaffold will help retain the cells at implantation site providing a supportive niche for the cells to proliferate, differentiate and make new cartilage and iv) the cells will secrete factors to recruit other cells to the implantation site to support cartilage regeneration.

**Table 4:**
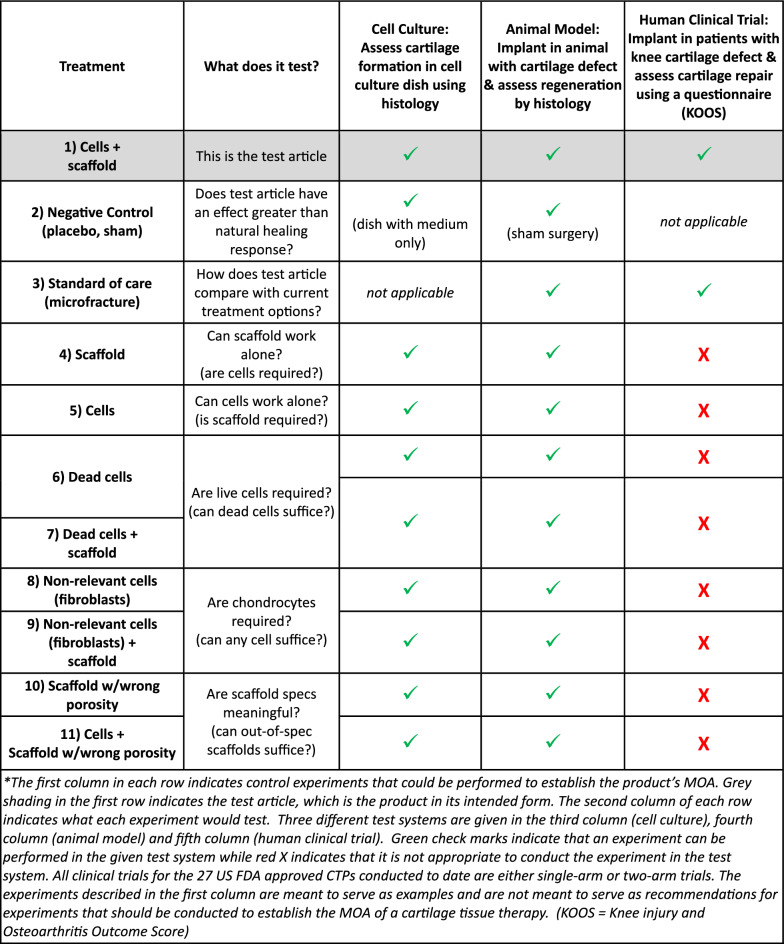
Example application for establishing MOA for a hypothetical product: Autologous cultured chondrocytes on collagen membrane for treating knee cartilage defects (based on MACI)

The test article is “Cells + scaffold” as shown in the first row of Table 4. The second row is “Negative control,” to assess if the test article has an effect above background levels. The negative control could be an untreated well for cell culture, a sham surgery for animals and may not be applicable for humans (since it may not be ethical to leave humans untreated). The third row is “Standard of care,” which is not applicable for cell culture, but could be microfracture for animals and humans. The fourth row is “Scaffold” only, which can establish if cells are required for the MOA. The fifth row is “Cells” only, which can establish if the cells by themselves can regenerate cartilage or if the scaffold is required. The sixth and seventh rows use dead cells instead of live cells, to establish that live cells are required for cartilage regeneration. The eighth and ninth rows use non-relevant cells, such as fibroblasts, to establish that the chondrocytes are required for the MOA. The tenth and eleventh rows use scaffolds with the wrong porosity (out of specification), to establish that scaffolds with the specified porosity are required for the MOA. All the experiments could be conducted in cells or animals, but only one, maybe two, can be conducted in humans. These examples demonstrate how clinical trials are not designed to establish the product’s MOA.

### Clinical trial structures for 27 CTPs approved in US

Table 2 (fourth column) shows the clinical trial structures (i.e., designs) for the 27 CTPs approved in the US. Only two of these 27 trials provide relevant insight on the MOA. Twenty one of the 27 trials (78%) were single-arm which cannot substantively inform the MOA. Six trials (22%) were two-arm, of which four did not shed much light on MOA: one compared to placebo (Laviv) and three were compared to standard of care (Gintuit, MACI, Stratagraft). One-arm trials, as well as two-arm trials that compare to placebo, untreated or standard of care, are useful for assessing efficacy, but shed little light on MOA. Thus, 25 of the 27 CTP trials (93%) did not substantively inform the MOA.

However, two of the 27 trials (7%) shed light on the MOA. The Provenge trial compared the test article, activated peripheral blood mononuclear cells (PBMCs), to unactivated PBMCs. This tested whether activated PBMCs were required for efficacy. Omisirge, umbilical cord blood (UCB) cells expanded ex vivo in the presence of nicotinamide, was compared to standard of care, which was untreated UCB cells. This tested whether cells expanded in the presence of nicotinamide performed comparably to unmanipulated UCB cells. Thus, only two of the clinical trials for the 27 approved CTPs had any real bearing on the MOA in humans.

### Control experiments in a research paper

To take the examples further, consider the challenge of achieving the evidence in a CTP clinical trial that would be required for a peer-reviewed basic research publication. Imagine if a research paper were submitted that reported only one experiment, the “cells + scaffold” experiment from Table 4, to assess cartilage regeneration in an animal model. Furthermore, imagine for this hypothetical study that no controls were run: no negative control, no cells only, no scaffold only, and no microfracture. The manuscript would likely be rejected with the reviewers requiring that the control experiments be conducted. Applying this same standard is neither feasible nor ethically responsible for CTP clinical trials, further illustrating the difficulty in establishing MOA in clinical trials.

### CTP clinical trials are designed to assess efficacy—not establish MOA

Given that only single-arm or two-arm clinical trials are conducted for CTPs, it may be unrealistic at this time to think that the MOA of a CTP could be established in humans with certainty. Indeed, CTP clinical trials are not designed to establish MOA but are instead designed to assess safety and efficacy. Even for cells and animals, where many types of experiments can be conducted (Tables 3 and 4), establishing the MOA is largely aspirational, as discussed above in the aspirin example. Aspirin affects at least five biochemical pathways which leads to uncertainty regarding its efficacy-relevant MOA in cells or animals.

## Considerations for the development of potency tests

### Potency test validation

Careful validation procedures for characterizing potency test performance are critical for assay reliability. Potency tests should be validated for specificity, sensitivity, linearity, range, accuracy, precision, repeatability (within lab variability), reproducibility (between lab variability), detection limit, quantification limit, robustness and fit for purpose [18, 59–61]. It should be established that the potency test can perform within defined specifications to help assure consistency in the manufactured product. Those specifications are set by measuring reference samples under a variety of conditions, modifying the assay to reduce uncertainty, and qualifying the assay response with respect to its precision and accuracy. The resulting criteria that are established for precision and accuracy in measurement of the reference sample are specifications that must be met when running the assay with the reference sample in parallel with the CTP sample.

### Matrix approach to potency tests

Use of several different potency tests, or a potency assay matrix [62], may be useful for CTPs for several reasons. First, CTPs likely have multiple MOAs. Second, there may be multiple product attributes that could be used as a potency attribute for any one of the MOAs for a given product. As discussed for a CAR T-cell therapy, its potency attribute could be defined as its ability to secrete IFN-γ upon binding to target cells (Fig. 2b), its ability to secrete IL5 upon binding to target cells, the ability of the CAR T-cells to kill target cells or a complex response that involves all three activities. Third, there may be a variety of ways to measure a given potency attribute. As discussed above, CAR T-cell release of IFN-γ release could be measured by ELISA or Western blot.

An assay matrix is also useful for the application of orthogonal methods for measuring a potency attribute in order to improve measurement confidence. Orthogonal measurements use different physical principles to measure the same property of the same sample with the goal of detecting method-specific biases and interferences [63]. The comparison of orthogonal methods can establish confidence in the accuracy of an assay. An example could be measurement of the ability of CAR T-cells to kill CD19-expressing cells by chromium release of target cells and by impedance plates (Fig. 2c). These measurements are orthogonal to one another because they are based on different physical principles: chromium release monitors cell health by assessing membrane integrity while impedance plates monitor cell health by the cells’ ability to adhere to the plate (since impedance decreases when cells detach). When a similar measurement result is obtained from the two techniques, confidence in the results is enhanced.

### Potency test variability, product variability, efficacy endpoint test variability and patient variability

It is difficult to determine if variability in potency test results is due to variability inherent in the test measurement, in the product or both. There may be variability in the biological reagents used for the potency test, such as the target cells in an IFN-γ release assay or in a cell-killing assay. There is well-known variability in antibody-based detection, such as detection of a secreted protein, like IFN-γ, by an ELISA. Assay validation strives to control for these variables with additional measurements that establish criteria for reagent storage and handling. There may be variability in the CTP, both within a batch or between batches.

Another challenge is variability in the patient population receiving the treatment. Patients in a population may vary from one another due to inherent biological, environmental and clinical factors, including previous treatments. Each patient’s condition may require different functionalities (*i.e.,* MOAs) from the product. Finally, there is variability in the efficacy endpoint testing, such as determining the amount of lymphoblasts in bone marrow [30] for a CAR T-cell clinical trial, that make it challenging to assess efficacy. Reducing variability in the product itself, the product manufacturing process, product testing (e.g., potency test), and efficacy endpoint tests, makes it more likely that a statistically significant clinical benefit to patients can be detected in a clinical trial. Reducing patient variability, by narrowing the indication, or refining the inclusion and exclusion criteria, may also increase the likelihood of detecting efficacy in a clinical trial.

### Short product shelf life

Short product shelf life may limit the availability of potency test results at the point of product release if the product has a shelf life that is shorter than the time it takes to conduct the potency test. These cases require rapid alternative methods of obtaining equivalent data for release. If a compendial method is required and that assay exceeds the shelf-life, a two-stage release may be appropriate using an alternative rapid assay for initial release followed by confirmation of the batch disposition by a validated, longer-term test.

### Stability and manufacturing changes

A potency test ought to be sensitive to product stability [7], such that products that have degraded from aging should demonstrate low potency test results. When changes are made to a manufacturing process, potency tests are important for assessing comparability between newer and older lots to assess how the changes may affect product performance [64].

### Potency assurance strategy

FDA recently released a new draft guidance for a potency assurance strategy for cell and gene therapy products [8]. “A potency assurance strategy is a multifaceted approach that reduces risks to the potency of a product through manufacturing process design, manufacturing process control, material control, in-process testing and potency lot release assays.” The new guidance emphasizes a lifecycle approach to potency that is grounded in quality risk management, where potency tests are considered throughout the product lifecycle from product development to licensure [65]. A lifecycle approach could allow the potency tests to change during product development as knowledge of the MOA, potency tests and risks to product potency is gained.

### Standards for potency tests

An ASTM “Standard Guide for Cell Potency Assays for Cell Therapy and Tissue Engineered Products” was published in 2019 that summarized current perspectives on potency from FDA Guidance Documents, US Pharmacopeia (USP), International Conference on Harmonization (ICH) and European Medicines Agency (EMA) [61]. A feasibility study on potency published in 2022 found that potency tests may not yet be ready for standards development [5]. However, some assays may be ready for development into standard test methods [66], such as CAR T-cell potency tests, cell viability tests and methods for quantifying the fraction of viable stems cells in a cell preparation. Of the six CAR T-cell therapies on the market, three of them reveal IFN-γ secretion and CAR expression as potency tests. In addition, 16 of the 27 approved cell therapies (59%) cite cell viability tests as a potency test (Table 2). These numbers may be higher, since many potency tests are redacted. The use of a cell viability test as a CTP potency test may not be ideal, since cell viability tests may not be specific enough with regard to the MOA. There are many CAR T-cell therapies and other types of cell therapies under development that might benefit from standard test methods for IFN-γ secretion, CAR expression and cell viability. Finally, methods for quantifying the specific fraction of viable tissue stem cells in cell preparations are under development as standard test methods for assessing the potency of stem cell therapy products [67]. Standard test methods should be vetted by interlaboratory studies to identify sources of variability and to assess reproducibility when the tests are conducted in different labs, by different operators and with different equipment [68].

### The future of MOA and potency tests

New methods for determining the MOA of CTPs may be developed in the future. The biological activity assessed in potency tests may be the result of complex interactions. Multi-omics is a promising approach where many omics modalities are used to collect data from the product and patients such as genomics, transcriptomics, proteomics, secretomics, metabolomics and lipidomics [69–71]. Design of experiments and multifactorial experimental designs hold promise for determining how complex biological processes operate [72, 73]. Machine learning and artificial intelligence may be useful for determining MOA and potency attributes from omics data. Systems level thinking, such as computational systems modeling, which takes a top-down approach that focuses on the macroscopic behavior of complex systems to predict behavior, may be useful for predicting the non-linear and emergent behavior of biological systems [74]. Automation and robotics for accelerated and higher throughput testing may also be important [75]. Collection of large amounts of product data and patient data during clinical trials will be helpful, so that the relationship between product attributes and patient attributes can be used to improve understanding of MOAs. It would be helpful if some of these efforts occur in the public domain, so that the data are publicly available [6] (Fig. 1). This may require industrial consortia due to the high costs and amount of effort that are required for clinical studies. Public domain product and patient data would allow the data science community at large to participate in developing innovative analytical methods for determining MOAs.

## Potency and MOA are useful concepts

Despite the challenges associated with MOA, potency, and efficacy that are discussed herein, potency tests and efforts to establish an MOA are important for CTPs. Human CTP clinical trials should have a rational and scientific underpinning where a sensible MOA is used as the motivation for product development and human investigation. Having an MOA and potency test that can withstand scientific scrutiny assures that human testing is conducted with a sound basis in scientific reasoning. Biologics are used for their biological activity and it makes sense to assess the quality of a CTP using a potency test that assesses its biological activity. This perspective offers a framework for interpreting what has been experienced for the US-approved CTPs. This perspective does not suggest that clinical trials should have multiple arms that include more of the experiments shown in Tables 3 and 4. The limitations of clinical trials may feel restrictive from a scientific standpoint, but these constraints are necessary to protect human subjects. The field of CTPs is rapidly evolving and the responses to the challenges presented herein will undoubtedly need to evolve as well.

## Conclusions

There are several key takeaways from this perspective:

- A measurand is “the quantity or property *intended* to be measured”, reminding us that all measurements have false positives and false negatives.
- A product attribute is often a measurand and should be defined independently from the measurement of the attribute.
- Potency ≠ efficacy (ideally, potency test results correlate with efficacy endpoint test results)
- A CTP can be “potent but not efficacious” or “not potent but efficacious.” The CTP development goal is to achieve “potent and efficacious.”
- Clinical trials are not designed to establish an MOA or validate a potency test; instead, clinical trials are designed to assess efficacy and safety.
- The clinical trials for the 27 US-approved CTPs were one- or two-arm trials; it is challenging to establish the MOA in humans or validate a potency test with any level of certainty with only one or two arms.
- Potency tests and efforts to establish an MOA are essential, since clinical trials should have a rational and scientific basis in a plausible MOA that guides product development and human investigation.

MOA, potency, potency test, efficacy, efficacy endpoint and efficacy endpoint tests should be independently defined to improve clarity during discussions concerning correlations between potency test results and clinical outcomes. Clarification of these independent terms will help to avoid hidden assumptions that result when concepts such as potency and efficacy are conflated. The ideas and observations presented herein may be helpful to product developers for setting realistic goals for understanding a product’s MOA and for establishing correlations between potency tests and clinical efficacy.

### Supplementary Information

**Additional file 1.** Key aspects for each of the 27 US-approved cell therapy products are summarized including product name, year approved, sponsor, product description, indication, clinical trial structure, efficacy endpoints, mechanism of action, potency test, comments and references.

**Additional file 2.** Key definitions that are referred to in the main text.

## Data Availability

Not applicable.

## Abbreviations

ALL: Acute lymphoblastic leukemia
CTP: Cell therapy product
CAR: Chimeric antigen receptor
COX: Cyclooxygenase
ELISA: Enzyme linked immunosorbent assay
EMA: European Medicines Agency
FDA: Food and Drug Administration
IFN-γ: Interferon-γ
ICH: International Conference on Harmonization
MOA: Mechanism of action
NF-κB: Nuclear factor kappa-light-chain-enhancer of activated B cells
PAP-GM-CSF: Human prostatic acid phosphatase (PAP) linked to human granulocyte–macrophage colony-stimulating factor
PBMC: Peripheral blood mononuclear cells
PCR: Polymerase chain reaction
SBRA: Summary Basis for Regulatory Action
US: United States
USP: US Pharmacopeia
VIM: Vocabulary of Metrology
