# Supplementary material for: Mechanism of action, potency and efficacy: considerations for cell therapies
Source: J Transl Med. 2024 May 2;22:416. doi: 10.1186/s12967-024-05179-7 (PMC11067168; doi:10.1186/s12967-024-05179-7)
Supplement: Supplementary file 1 — Additional file 1. Key aspects for each of the 27 US-approved cell therapy products are summarized including product name, year approved, sponsor, product description, indication, clinical trial structure, efficacy endpoints, mechanism of action, potency test, comments and references. [file 12967_2024_5179_MOESM1_ESM.pdf]

Supplementary File 1

Simon et al., Mechanism of Action, Potency and Efficacy: Considerations for Cell **Therapies**

Cell Therapy Products Approved by FDA for Marketing in the USA

Summary: The following pages contain a 1-page summary of each of the 27 cell therapy products (CTPs) approved for marketing in the US. Key aspects of each product are summarized including product name, year approved, sponsor, product description, indication, clinical trial structure, efficacy endpoints, mechanism of action, potency test, comments and references. Two cell-containing products are omitted from this list, Apligraf (approved 1998) and Dermagraf (approved 2001), because they were reviewed by the US FDA as devices and potency tests were not required. A key resource is an FDA webpage that provides regulatory documentation for the 27 approved CTPs: https://www.fda.gov/vaccines-blood-biologics/cellular-gene-therapy-products/approved-cellular-and-gene-therapy-products.

Kymriah (tisagenlecleucel, Novartis Pharmaceuticals)....2
Yescarta (axicabtagene ciloleucel, Kite Pharma).... 3
Tecartus (brexucabtagene autoleucel, Kite Pharma) 4
Breyanzi (lisocabtagene maraleucel, Juno Therapeutics) 5
Abecma (idecabtagene vicleucel, Celgene)....6
Carvykti (ciltacabtagene autoleucel, Janssen Biotech)....7
Hemacord (New York Blood Center) 8
HPC Cord Blood (Clinimmune Labs, University of Colorado Cord Blood Bank)....9
Ducord (Duke University School of Medicine, Carolinas Cord Blood Bank).... 10
HPC Cord Blood (LifeSouth Community Blood Centers).... 11
HPC Cord Blood (Bloodworks).... 12
Allocord (SSM Cardinal Glennon Children's Medical Center).... 13
Clevecord (Cleveland Cord Blood Center).... 14
HPC Cord Blood (MD Anderson Cord Blood Bank).... 15
Provenge (sipuleucel-T, Dendreon).... 16
Laviv (Azficel-T, Fibrocell Technologies).... 17
Gintuit (Organogenesis).... 18
MACI (matrix-induced autologous chondrocyte implantation, Vericel).... 19
Stratagraft (Stratech).... 20
Rethymic (Enzyvant Therapeutics).... 21
Zynteglo (betibeglogene autotemcel, Bluebird Bio).... 22
Skysona (elivaldogene autotemcel, Bluebird Bio).... 23
Omisirge (omidubicel, Gamida Cell Ltd.).... 24
Lantidra (donislecel-jujn, CellTrans Inc.).... 25
Casgevy (exagamglogene autotemcel, Vertex Pharmaceuticals Inc.).... 26
Lyfgenia (lovotibeglogene autotemcel, Bluebird Bio).... 27
Amtagvi (lifileucel, Iovance Biotherapeutics, Inc).... 28

Product: Kymriah (tisagenlecleucel) **Year approved:** 2017

Sponsor: Novartis Pharmaceuticals

Description: "KYMRIAH™ (tisagenlecleucel) is a CD19-directed genetically modified autologous T cell immunotherapy comprised of autologous T cells that are genetically modified using a lentiviral vector to encode an anti-CD19 chimeric antigen receptor (CAR). The CAR is comprised of a murine single-chain antibody fragment (scFv) specific for CD19, followed by a CD8 hinge and transmembrane region that is fused to the intracellular signaling domains for 4-1BB (CD137) and CD3 zeta. KYMRIAH is prepared from the patient's peripheral blood mononuclear cells, which are obtained via a standard leukapheresis procedure. The mononuclear cells are enriched for T cells, then transduced with the lentiviral vector containing the anti-CD19 CAR transgene, and activated with anti-CD3/CD28 antibody coated beads. The transduced T cells are expanded in cell culture, washed, and formulated into a suspension, which then is cryopreserved. The product must pass a sterility test before release for shipping as a frozen suspension in a patient-specific infusion bag(s). The product is thawed prior to administration [see Dosage and Administration (2.3), How Supplied/Storage and Handling (16)]. The thawed product is a colorless to slightly yellow suspension of cells."

Indication: Indicated for the treatment of patients up to 25 years of age with B-cell precursor acute lymphoblastic leukemia (ALL) that is refractory or in second or later relapse.

Clinical Trial Structure: Single arm; compared to historical data for standard of care

Efficacy Endpoints: Overall remission rate (complete remission defined as <5% lymphoblasts in bone marrow by morphology, circulating blasts <1% in peripheral blood, no evidence of extramedullary disease, neutrophils >1.0×10^9/L, platelets >100×109/L, and no platelet and/or neutrophil transfusions within 7 days of peripheral blood sample for disease assessment)

MOA (Package Insert): "KYMRIAH is a CD19-directed genetically modified autologous T-cell immunotherapy which involves reprogramming a patient's own T-cells with a transgene encoding a chimeric antigen receptor (CAR) to identify and eliminate CD19-expressing malignant and normal cells. The CAR is comprised of a murine single-chain antibody fragment which recognizes CD19 and is fused to intracellular signaling domains from 4-1BB (CD137) and CD3 zeta. The CD3 zeta component is critical for initiating T-cell activation and antitumor activity, while 4-1BB enhances the expansion and persistence of KYMRIAH. Upon binding to CD19-expressing cells, the CAR transmits a signal to promote T-cell expansion, activation, target cell elimination, and persistence of the KYMRIAH cells."

Potency Testing (FDA Panel Meeting Slides): i) Determination of CAR expression by flow cytometry; ii) Interferon-γ production by product upon stimulation with CD19+ cells

Potency Comments (FDA Briefing Document): "In the clinical trials, IFN-γ production varied greatly from lot-to-lot, making it difficult to correlate IFN-γ production in vitro to tisagenlecleucel safety or efficacy."

References

- Summary Basis for Regulatory Action - Kymriah, FDA, 2017. Accessed May 2, 2023: https://www.fda.gov/vaccines-blood-biologics/cellular-gene-therapy-products/kymriah-tisagenlecleucel
- Package Insert - Kymriah, FDA, 2017. Accessed April 27, 2023: https://www.fda.gov/vaccines-blood-biologics/cellular-gene-therapy-products/kymriah-tisagenlecleucel
- Maude SL, Laetsch TW, Buechner J, Rives S, Boyer M, Bittencourt H, Bader P, Verneris MR, Stefanski HE, Myers GD, Qayed M, De Moerloose B, Hiramatsu H, Schlis K, Davis KL, Martin PL, Nemecek ER, Yanik GA, Peters C, Baruchel A, Boissel N, Mechinaud F, Balduzzi A, Krueger J, June CH, Levine BL, Wood P, Taran T, Leung M, Mueller KT, Zhang Y, Sen K, Lebwohl D, Pulsipher MA, Grupp SA. Tisagenlecleucel in Children and Young Adults with B-Cell Lymphoblastic Leukemia. N Engl J Med 2018 378(5):439-448. https://doi.org/10.1056/NEJMoa1709866
- Slides for the July 12, 2017 Meeting of the Oncologic Drugs Advisory Committee (ODAC). Novartis Slides, FDA, 2017. Accessed May 17, 2023: https://www.fda.gov/media/106496/download
- FDA Briefing Document for the July 12, 2017 Oncologic Drugs Advisory Committee Meeting, FDA, 2017. Accessed May 17, 2023: https://www.fda.gov/media/106081/download

Product: Yescarta (axicabtagene ciloleucel) **Year approved:** 2017

Sponsor: Kite Pharma

Description: Genetically modified, antigen-specific autologous T-cells reprogrammed to target cells that express CD19

Indication: For the treatment of adult patients with relapsed or refractory large B-cell lymphoma after two or more lines of systemic therapy, including diffuse large B-cell lymphoma (DLBCL) not otherwise specified, primary mediastinal large B-cell lymphoma, high-grade B-cell lymphoma, and DLBCL arising from follicular lymphoma

Clinical Trial Structure: Single arm (compared to historical data for standard of care)

Efficacy Endpoints: Complete remission (as defined complete disappearance of all detectable clinical evidence of disease and symptoms; typically assessed by absence of masses by positron emission tomography (PET), computed tomography (CT) to assess absence of liver/spleen enlargement, bone marrow biopsy)

MOA (package insert): "YESCARTA, a CD19-directed genetically modified autologous T-cell immunotherapy, binds to CD19-expressing cancer cells and normal B cells. Studies demonstrated that following anti-CD19 CAR T-cell engagement with CD19-expressing target cells, the CD28 and CD3-zeta co-stimulatory domains activate downstream signaling cascades that lead to T-cell activation, proliferation, acquisition of effector functions and secretion of inflammatory cytokines and chemokines. This sequence of events leads to killing of CD19-expressing cells."

Potency Testing:

- *FDA SBRA*: i) Cell viability; ii) anti-CD19 CAR expression; iii) redacted
- *Papadouli et al., 2020:* Interferon-γ production by product upon stimulation with CD19+ cells

References

- Summary Basis for Regulatory Action - Yescarta, FDA, 2017. Accessed April 27, 2023: https://www.fda.gov/vaccines-blood-biologics/cellular-gene-therapy-products/yescarta-axicabtagene-ciloleucel
- Package Insert - Yescarta, FDA, 2017. Accessed April 27, 2023: https://www.fda.gov/vaccines-blood-biologics/cellular-gene-therapy-products/yescarta-axicabtagene-ciloleucel
- Neelapu SS, Locke FL, Bartlett NL, Lekakis LJ, Miklos DB, Jacobson CA, Braunschweig I, Oluwole OO, Siddiqi T, Lin Y, Timmerman JM, Stiff PJ, Friedberg JW, Flinn IW, Goy A, Hill BT, Smith MR, Deol A, Farooq U, McSweeney P, Munoz J, Avivi I, Castro JE, Westin JR, Chavez JC, Ghobadi A, Komanduri KV, Levy R, Jacobsen ED, Witzig TE, Reagan P, Bot A, Rossi J, Navale L, Jiang Y, Aycock J, Elias M, Chang D, Wiezorek J, Go WY. Axicabtagene Ciloleucel CAR T-Cell Therapy in Refractory Large B-Cell Lymphoma. N Engl J Med 2017;377(26):2531-2544. https://doi.org/10.1056/NEJMoa1707447
- Papadouli I, Mueller-Berghaus J, Beuneu C, Ali S, Hofner B, Petavy F, Tzogani K, Miermont A, Norga K, Kholmanskikh O, Leest T, Schuessler-Lenz M, Salmonson T, Gisselbrecht C, Garcia JL, Pignatti F. EMA Review of Axicabtagene Ciloleucel (Yescarta) for the Treatment of Diffuse Large B-Cell Lymphoma. Oncologist 2020;25(10):894-902. https://doi.org/10.1634/theoncologist.2019-0646

Product: Tecartus (brexucabtagene autoleucel) **Year approved:** 2020

Sponsor: Kite Pharma

Description: TECARTUS is comprised of genetically modified, antigen-specific autologous T-cells reprogrammed to target cells that express CD19, an antigen expressed on the surface of healthy and malignant B cells. The TECARTUS chimeric antigen receptor (CAR) protein has a murine single chain variable fragment (scFv) specific to human CD19 linked to two signaling domains derived from human CD28 and CD3ζ. The CAR protein plays a critical role in TECARTUS function, mediating T-cell activation and anti-tumor effector function following binding of the scFv to CD19. The CAR expressed in TECARTUS is identical to that in YESCARTA (axicabtagene ciloleucel), a CD19-directed genetically modified autologous T-cell immunotherapy approved in 2017 for relapsed or refractory large B cell lymphoma. TECARTUS differs from YESCARTA in that T-cells are enriched during the TECARTUS manufacturing process; T-cell enrichment is not performed during YESCARTA manufacture.

Indication: Relapsed/refractory mantle cell lymphoma

Clinical Trial Structure: Single arm (compared to historical data for standard of care)

Efficacy Endpoint: Objective response rate [typically assessed by positron emission tomography-computed tomography (PET-CT) for presence of lesions, bone marrow biopsy, lumbar puncture for examination of cerebral spinal fluid (CSF)]

MOA (package insert): "TECARTUS, a CD19-directed genetically modified autologous T-cell immunotherapy, binds to CD19-expressing cancer cells and normal B cells. Studies demonstrated that following anti-CD19 CAR T-cell engagement with CD19-expressing target cells, the CD28 and CD3-zeta co-stimulatory domains activate downstream signaling cascades that lead to T-cell activation, proliferation, acquisition of effector functions, and secretion of inflammatory cytokines and chemokines. This sequence of events leads to killing of CD19-expressing cells."

Potency Testing (FDA SBRA): i) Cell viability; ii) anti-CD19 CAR expression; iii) redacted

References

- Summary Basis for Regulatory Action - Tecartus, FDA, 2020. Accessed April 27, 2023: https://www.fda.gov/vaccines-blood-biologics/cellular-gene-therapy-products/tecartus-brexucabtagene-autoleucel
- Package Insert - Tecartus, FDA, 2020. Accessed April 27, 2023: https://www.fda.gov/vaccines-blood-biologics/cellular-gene-therapy-products/tecartus-brexucabtagene-autoleucel
- Wang M, Munoz J, Goy A, Locke FL, Jacobson CA, Hill BT, Timmerman JM, Holmes H, Jaglowski S, Flinn IW, McSweeney PA, Miklos DB, Pagel JM, Kersten MJ, Milpied N, Fung H, Topp MS, Houot R, Beitinjaneh A, Peng W, Zheng L, Rossi JM, Jain RK, Rao AV, Reagan PM. KTE-X19 CAR T-Cell Therapy in Relapsed or Refractory Mantle-Cell Lymphoma. N Engl J Med 2020;382(14):1331-1342. https://doi.org/10.1056/NEJMoa1914347
- Assessment Report, European Medicines Agency, Committee for Medicinal Products for Human Use, 2020. Accessed May 2, 2023: https://www.ema.europa.eu/en/documents/assessment-report/tecartus-epar-public-assessment-report_en.pdf

Product: Breyanzi (lisocabtagene maraleucel) **Year approved:** 2021

Sponsor: Juno Therapeutics

Description: CD19-directed genetically modified autologous T-cell immunotherapy

Indication: Adult patients with relapsed or refractory large B-cell lymphoma after two or more lines of systemic therapy, including diffuse large B-cell lymphoma (DLBCL) not otherwise specified (including DLBCL arising from indolent lymphoma), high-grade B-cell lymphoma, primary mediastinal large B-cell lymphoma, and follicular lymphoma grade 3B

Clinical Trial Structure: Single arm (with 3 dose cohorts) (compared to historical data for standard of care)

Efficacy Endpoints: Objective response rate as assessed by i) survival, ii) disease progression

MOA (Package Insert): "CAR binding to CD19 expressed on the cell surface of tumor and normal B cells induces activation and proliferation of CAR T-cells, release of pro-inflammatory cytokines, and cytotoxic killing of target cells."

Potency Testing (FDA SBRA): i) Redacted

References

- Summary Basis for Regulatory Action - Breyanzi, FDA, 2021. Accessed April 27, 2023: https://www.fda.gov/vaccines-blood-biologics/cellular-gene-therapy-products/breyanzi-lisocabtagene-maraleucel
- Package Insert - Breyanzi, FDA, 2021. Accessed April 27, 2023: https://www.fda.gov/vaccines-blood-biologics/cellular-gene-therapy-products/breyanzi-lisocabtagene-maraleucel
- Abramson JS, Palomba ML, Gordon LI, Lunning MA, Wang M, Arnason J, Mehta A, Purev E, Maloney DG, Andreadis C, Sehgal A, Solomon SR, Ghosh N, Albertson TM, Garcia J, Kostic A, Mallaney M, Ogasawara K, Newhall K, Kim Y, Li D, Siddiqi T. Lisocabtagene maraleucel for patients with relapsed or refractory large B-cell lymphomas (TRANSCEND NHL 001): a multicentre seamless design study. Lancet 2020;396(10254):839-852. https://doi.org/10.1016/S0140-6736(20)31366-0

Product: Abecma (idecabtagene vicleucel) **Year approved:** 2021

Sponsor: Celgene

Description: B-cell maturation antigen (BCMA)-directed genetically modified autologous T-cell immunotherapy

Indication: Adult patients with relapsed or refractory multiple myeloma after four or more prior lines of therapy, including an immunomodulatory agent, a proteasome inhibitor, and an anti-CD38 monoclonal antibody

Clinical Trial Structure: Single arm (with 3 dose cohorts) (compared to historical data for standard of care)

Efficacy Endpoints: Objective response rate as defined by i) serum and urine protein electrophoresis and immunofixation, ii) serum immunoglobulins (IgG, IgM, and IgA), iii) serum free light chain assay, iv) serum chemistry for creatinine, v) radiographic assessment of bone lesions, vi) extramedullary plasmacytoma assessments [by positron emission tomography-computed tomography (PET-CT) or magnetic resonance imaging (MRI)], vii) bone marrow biopsy (CD138+ cells and BCMA expression)

MOA (Package Insert): "ABECMA is a chimeric antigen receptor (CAR)-positive T-cell therapy targeting B-cell maturation antigen (BCMA), which is expressed on the surface of normal and malignant plasma cells. The CAR construct includes an anti-BCMA scFv-targeting domain for antigen specificity, a transmembrane domain, a CD3-zeta T-cell activation domain, and a 4-1BB costimulatory domain. Antigen-specific activation of ABECMA results in CAR-positive T-cell proliferation, cytokine secretion, and subsequent cytolytic killing of BCMA-expressing cells."

Potency Testing

- *FDA SBRA:* i) Redacted
- *EMA Assessment Report:* Interferon-γ production by product upon stimulation with BCMA+ cells

References

- Summary Basis for Regulatory Action - Abecma, FDA, 2021. Accessed May 2, 2023: https://www.fda.gov/vaccines-blood-biologics/abecma-idecabtagene-vicleucel
- Package Insert - Abecma, FDA, 2021. Accessed May 2, 2023: https://www.fda.gov/vaccines-blood-biologics/abecma-idecabtagene-vicleucel
- Munshi NC, Anderson LD Jr, Shah N, Madduri D, Berdeja J, Lonial S, Raje N, Lin Y, Siegel D, Oriol A, Moreau P, Yakoub-Agha I, Delforge M, Cavo M, Einsele H, Goldschmidt H, Weisel K, Rambaldi A, Reece D, Petrocca F, Massaro M, Connarn JN, Kaiser S, Patel P, Huang L, Campbell TB, Hege K, San-Miguel J. Idecabtagene Vicleucel in Relapsed and Refractory Multiple Myeloma. N Engl J Med. 2021;384(8):705-716. https://doi.org/10.1056/NEJMoa2024850
- Assessment Report, European Medicines Agency, Committee for Medicinal Products for Human Use, 2021. Accessed May 2, 2023: https://www.ema.europa.eu/en/documents/assessment-report/abecma-epar-public-assessment-report_en.pdf

Product: Carvykti (ciltacabtagene autoleucel) **Year approved:** 2023

Sponsor: Janssen Biotech

Description: B-cell maturation antigen (BCMA)-directed genetically modified autologous T-cell immunotherapy

Indication: Adult patients with relapsed or refractory multiple myeloma after four or more prior lines of therapy, including a proteasome inhibitor, an immunomodulatory agent, and an anti-CD38 monoclonal antibody

Clinical Trial Structure: Single arm (compared to historical data for standard of care)

Efficacy Endpoints: Survival and objective response rate (as defined by negative response for disease evaluations: i) myeloma protein measurements in serum and urine; ii) serum calcium corrected for albumin; iii) bone marrow examination; iv) skeletal survey; v) documentation of extramedullary plasmacytomas)

MOA (Package Insert): "CARVYKTI is a BCMA-directed, genetically modified autologous T-cell immunotherapy, which involves reprogramming a patient's own T-cells with a transgene encoding a chimeric antigen receptor (CAR) that identifies and eliminates cells that express BCMA. The CARVYKTI CAR protein features two BCMA-targeting single-domain antibodies designed to confer high avidity against human BCMA, a 4-1BB co-stimulatory domain and a CD3-zeta (CD3ζ) signaling cytoplasmic domain. Upon binding to BCMA-expressing cells, the CAR promotes T-cell activation, expansion, and elimination of target cells."

Potency Testing (FDA SBRA): i) CAR expression from viable T-cells; ii) redacted

References

- Summary Basis for Regulatory Action - Carvykti, FDA, 2023. Accessed April 25, 2023: https://www.fda.gov/vaccines-blood-biologics/carvykti
- Package Insert – Carvykti, FDA, 2023. Accessed April 25, 2023: https://www.fda.gov/vaccines-blood-biologics/carvykti

Product: Hemacord **Year approved:** 2011

Sponsor: New York Blood Center

Description: Allogeneic cord blood hematopoietic progenitor cell therapy

Indication: Unrelated donor hematopoietic progenitor cell transplantation procedures in conjunction with an appropriate preparative regimen for hematopoietic and immunologic reconstitution in patients with disorders affecting the hematopoietic system that are inherited, acquired, or result from myeloablative treatment.

Clinical Trial Structure: Single arm (compared to historical data for standard of care)

Efficacy Endpoints: Hematologic reconstitution (neutrophils, platelets, erythrocytes)

MOA (Package Insert): "Hematopoietic stem/progenitor cells from HPC, Cord Blood migrate to the bone marrow where they divide and mature. The mature cells are released into the bloodstream, where some circulate and others migrate to tissue sites, partially or fully restoring blood counts and function, including immune function, of blood-borne cells of marrow origin."

Potency Testing (FDA SBRA): i) Total nucleated cell number; ii) viability of CD45+ cells; iii) viable CD34+ cell count; iv) Colony forming unit (CFU)

References

- Summary Basis for Regulatory Action - Hemacord, FDA, 2011. Accessed April 27, 2023: https://www.fda.gov/vaccines-blood-biologics/cellular-gene-therapy-products/hemacord-hpc-cord-blood
- Package Insert - Hemacord, FDA, 2011. Accessed April 27, 2023: https://www.fda.gov/vaccines-blood-biologics/cellular-gene-therapy-products/hemacord-hpc-cord-blood

Product: HPC Cord Blood - Clinimmune Labs **Year approved:** 2012

Sponsor: University of Colorado Cord Blood Bank

Description: Allogeneic cord blood hematopoietic progenitor cell therapy

Indication: Unrelated donor hematopoietic progenitor cell transplantation procedures in conjunction with an appropriate preparative regimen for hematopoietic and immunologic reconstitution in patients with disorders affecting the hematopoietic system that are inherited, acquired, or result from myeloablative treatment.

Clinical Trial Structure: Single arm (compared to historical data for standard of care)

Efficacy Endpoints: Hematologic reconstitution (neutrophils, platelets, erythrocytes)

MOA (Package Insert): "Hematopoietic stem/progenitor cells from HPC, Cord Blood migrate to the bone marrow where they divide and mature. The mature cells are released into the bloodstream, where some circulate and others migrate to tissue sites, partially or fully restoring blood counts and function, including immune function, of blood-borne cells of marrow origin."

Potency Testing (FDA SBRA): i) Total nucleated cells; ii) viability of total nucleated cells; iii) viable CD34+ cell count; iv) redacted

References

- Summary Basis for Regulatory Action, HPC Cord Blood - Clinimmune Labs, FDA, 2012. Accessed April 27, 2023: https://www.fda.gov/vaccines-blood-biologics/cellular-gene-therapy-products/hpc-cord-blood
- Package Insert - HPC Cord Blood - Clinimmune Labs, FDA, 2012. Accessed April 27, 2023: https://www.fda.gov/vaccines-blood-biologics/cellular-gene-therapy-products/hpc-cord-blood

Product: Ducord **Year approved:** 2012

Sponsor: Duke University School of Medicine, Carolinas Cord Blood Bank

Description: Allogeneic cord blood hematopoietic progenitor cell therapy

Indication: Unrelated donor hematopoietic progenitor cell transplantation procedures in conjunction with an appropriate preparative regimen for hematopoietic and immunologic reconstitution in patients with disorders affecting the hematopoietic system that are inherited, acquired, or result from myeloablative treatment.

Clinical Trial Structure: Single arm (compared to historical data for standard of care)

Efficacy Endpoint: Hematologic reconstitution (neutrophils, platelets, erythrocytes)

MOA (Package Insert): "Hematopoietic stem/progenitor cells from HPC, Cord Blood migrate to the bone marrow where they divide and mature. The mature cells are released into the bloodstream, where some circulate and others migrate to tissue sites, partially or fully restoring blood counts and function, including immune function, of blood-borne cells of marrow origin."

Potency Testing (FDA SBRA): i) Total nucleated cells; ii) viable nucleated cells; iii) viable CD34+ cells (flow cytometry); iv) redacted; v) redacted

References

- Summary Basis for Regulatory Action - Ducord, FDA, 2012. Accessed April 27, 2023: https://www.fda.gov/vaccines-blood-biologics/cellular-gene-therapy-products/ducord-hpc-cord-blood
- Package Insert - Ducord, FDA, 2012. Accessed April 27, 2023: https://www.fda.gov/vaccines-blood-biologics/cellular-gene-therapy-products/ducord-hpc-cord-blood

Product: HPC Cord Blood – LifeSouth **Year approved:** 2013

Sponsor: LifeSouth Community Blood Centers

Description: Allogeneic cord blood hematopoietic progenitor cell therapy

Indication: Unrelated donor hematopoietic progenitor cell transplantation procedures in conjunction with an appropriate preparative regimen for hematopoietic and immunologic reconstitution in patients with disorders affecting the hematopoietic system that are inherited, acquired, or result from myeloablative treatment.

Clinical Trial Structure: Single arm (compared to historical data for standard of care)

Efficacy Endpoints: Hematologic reconstitution (neutrophils, platelets, erythrocytes)

MOA (Package Insert): "Hematopoietic stem/progenitor cells from HPC, Cord Blood migrate to the bone marrow where they divide and mature. The mature cells are released into the bloodstream, where some circulate and others migrate to tissue sites, partially or fully restoring blood counts and function, including immune function, of blood-borne cells of marrow origin."

Potency Testing (FDA SBRA): i) Total nucleated cells; ii) viable nucleated cells; iii) viable CD34+ cells (flow cytometry); iv) redacted

References

- Summary Basis for Regulatory Action, HPC Cord Blood - LifeSouth, FDA, 2013. Accessed April 27, 2023: https://www.fda.gov/vaccines-blood-biologics/cellular-gene-therapy-products/hpc-cord-blood-lifesouth
- Package Insert - HPC Cord Blood - LifeSouth, FDA, 2013. Accessed April 27, 2023: https://www.fda.gov/vaccines-blood-biologics/cellular-gene-therapy-products/hpc-cord-blood-lifesouth

Product: HPC Cord Blood – Bloodworks **Year approved:** 2016

Sponsor: Bloodworks

Description: Allogeneic cord blood hematopoietic progenitor cell therapy

Indication: Unrelated donor hematopoietic progenitor cell transplantation procedures in conjunction with an appropriate preparative regimen for hematopoietic and immunologic reconstitution in patients with disorders affecting the hematopoietic system that are inherited, acquired, or result from myeloablative treatment.

Clinical Trial Structure: Single arm (compared to historical data for standard of care)

Efficacy Endpoints: Hematologic reconstitution (neutrophils, platelets, erythrocytes)

MOA (Package Insert): "Hematopoietic stem/progenitor cells from HPC, Cord Blood migrate to the bone marrow where they divide and mature. The mature cells are released into the bloodstream, where some circulate and others migrate to tissue sites, partially or fully restoring blood counts and function, including immune function, of blood-borne cells of marrow origin."

Potency Testing (FDA SBRA): i) Total nucleated cells; ii) viable nucleated cells; iii) viable CD34+ cells (flow cytometry); iv) redacted

References

- Summary Basis for Regulatory Action, HPC Cord Blood - Bloodworks, FDA, 2016. Accessed April 27, 2023: https://www.fda.gov/vaccines-blood-biologics/cellular-gene-therapy-products/hpc-cord-blood-bloodworks
- Package Insert - HPC Cord Blood - Bloodworks, FDA, 2016. Accessed April 27, 2023: https://www.fda.gov/vaccines-blood-biologics/cellular-gene-therapy-products/hpc-cord-blood-bloodworks

Product: Allocord **Year approved:** 2013

Sponsor: SSM Cardinal Glennon Children's Medical Center

Description: Allogeneic cord blood hematopoietic progenitor cell therapy

Indication: Unrelated donor hematopoietic progenitor cell transplantation procedures in conjunction with an appropriate preparative regimen for hematopoietic and immunologic reconstitution in patients with disorders affecting the hematopoietic system that are inherited, acquired, or result from myeloablative treatment.

Clinical Trial Structure: Single arm (compared to historical data for standard of care)

Efficacy Endpoints: Hematologic reconstitution (neutrophils, platelets, erythrocytes)

MOA (Package Insert): "Hematopoietic stem/progenitor cells from HPC, Cord Blood, migrate to the bone marrow where they divide and mature. The mature cells are released into the bloodstream, where some circulate and others migrate to tissue sites, partially or fully restoring blood counts and function, including immune function, of blood-borne cells of marrow origin."

Potency Testing (FDA SBRA): i) Total nucleated cells; ii) viable nucleated cells; iii) viable CD34+ cell count; iv) colony forming units (CFU)

References

- Summary Basis for Regulatory Action - Allocord, FDA, 2012. Accessed April 27, 2023: https://www.fda.gov/vaccines-blood-biologics/cellular-gene-therapy-products/allocord-hpc-cord-blood
- Package Insert - Allocord, FDA, 2013. Accessed April 27, 2023: https://www.fda.gov/vaccines-blood-biologics/cellular-gene-therapy-products/allocord-hpc-cord-blood

Product: Clevecord **Year approved:** 2016

Sponsor: Cleveland Cord Blood Center

Description: Allogeneic cord blood hematopoietic progenitor cell therapy

Indication: Unrelated donor hematopoietic progenitor cell transplantation procedures in conjunction with an appropriate preparative regimen for hematopoietic and immunologic reconstitution in patients with disorders affecting the hematopoietic system that are inherited, acquired, or result from myeloablative treatment.

Clinical Trial Structure: Single arm (compared to historical data for standard of care)

Efficacy Endpoints: Hematologic reconstitution (neutrophils, platelets, erythrocytes)

MOA (Package Insert): "Hematopoietic stem/progenitor cells from HPC, Cord Blood migrate to the bone marrow where they divide and mature. The mature cells are released into the bloodstream, where some circulate and others migrate to tissue sites, partially or fully restoring blood counts and function, including immune function, of blood-borne cells of marrow origin."

Potency Testing (FDA SBRA): i) Total nucleated cell number; ii) viable nucleated cells; iii) viable CD34+ cell count; iv) redacted

References

- Summary Basis for Regulatory Action (SBRA), Clevecord, FDA, 2016. Accessed April 27, 2023: https://www.fda.gov/vaccines-blood-biologics/cellular-gene-therapy-products/clevecord-hpc-cord-blood
- Package Insert - Clevecord, FDA, 2016. Accessed April 27, 2023: https://www.fda.gov/vaccines-blood-biologics/cellular-gene-therapy-products/clevecord-hpc-cord-blood
- Laughlin MJ, Barker J, Bambach B, Koc ON, Rizzieri DA, Wagner JE, Gerson SL, Lazarus HM, Cairo M, Stevens CE, Rubinstein P, Kurtzberg J. Hematopoietic engraftment and survival in adult recipients of umbilical-cord blood from unrelated donors. N Engl J Med. 2001 Jun 14;344(24):1815-22. https://doi.org/10.1056/NEJM200106143442402
- Laughlin MJ, Eapen M, Rubinstein P, Wagner JE, Zhang MJ, Champlin RE, Stevens C, Barker JN, Gale RP, Lazarus HM, Marks DI, van Rood JJ, Scaradavou A, Horowitz MM. Outcomes after transplantation of cord blood or bone marrow from unrelated donors in adults with leukemia. N Engl J Med. 2004 Nov 25;351(22):2265-75. https://doi.org/10.1056/NEJMoa041276
- Cornetta K, Laughlin M, Carter S, Wall D, Weinthal J, Delaney C, Wagner J, Sweetman R, McCarthy P, Chao N. Umbilical cord blood transplantation in adults: results of the prospective Cord Blood Transplantation (COBLT). Biol Blood Marrow Transplant 2005;11(2):149-60. https://doi.org/10.1016/j.bbmt.2004.11.020

Product: HPC Cord Blood - MD Anderson **Year approved:** 2018

Sponsor: MD Anderson Cord Blood Bank

Description: Allogeneic cord blood hematopoietic progenitor cell therapy

Indication: Unrelated donor hematopoietic progenitor cell transplantation procedures in conjunction with an appropriate preparative regimen for hematopoietic and immunologic reconstitution in patients with disorders affecting the hematopoietic system that are inherited, acquired, or result from myeloablative treatment.

Clinical Trial Structure: Single arm (compared to historical data for standard of care)

Efficacy Endpoints: Hematologic reconstitution (neutrophils, platelets, erythrocytes)

MOA (Package Insert): "Hematopoietic stem/progenitor cells from HPC, Cord Blood migrate to the bone marrow where they divide and mature. The mature cells are released into the bloodstream, where some circulate and others migrate to tissue sites, partially or fully restoring peripheral blood counts and function, including immune function, of blood-borne cells of marrow origin."

Potency Testing (FDA SBRA): i) Total CD34+ count; ii) total nucleated cell count; iii) nucleated red blood cell; iv) viability of nucleated cells; v) viable CD34+ cells; vi) colony forming units assay (CFU)

References

- Summary Basis for Regulatory Action (SBRA), HPC Cord Blood - MD Anderson, FDA, 2018. Accessed April 27, 2023: https://www.fda.gov/vaccines-blood-biologics/cellular-gene-therapy-products/hpc-cord-blood-md-anderson-cord-blood-bank
- Package Insert - HPC Cord Blood - MD Anderson, FDA, 2018. Accessed April 27, 2023: https://www.fda.gov/vaccines-blood-biologics/cellular-gene-therapy-products/hpc-cord-blood-md-anderson-cord-blood-bank

Product: Provenge (sipuleucel-T) **Year approved:** 2010

Sponsor: Dendreon

Description: Autologous cellular immunotherapy (CD54+ cells activated with PAP-GM-CSF and suspended in Ringer's)

Indication: Asymptomatic or minimally symptomatic metastatic castrate resistant (hormone refractory) prostate cancer

Clinical Trial Structure: Two arm (Provenge compared to control autologous peripheral blood mononuclear cells that have not been activated)

Efficacy Endpoints: Survival

MOA (Package Insert): "While the precise mechanism of action is unknown, PROVENGE is designed to induce an immune response targeted against PAP, an antigen expressed in most prostate cancers. During ex vivo culture with PAP-GM-CSF, APCs take up and process the recombinant target antigen into small peptides that are then displayed on the APC surface."

Potency Testing (FDA SBRA): i) Number of CD54+ cells (flow cytometry); ii) increased expression of CD54 on the surface of antigen presenting cells after culture with PAP-GM-CSF (flow cytometry)

References

- Summary Basis for Regulatory Action – Provenge, FDA, 2010. Accessed April 27, 2023: https://www.fda.gov/vaccines-blood-biologics/cellular-gene-therapy-products/provenge-sipuleucel-t
- Package Insert – Provenge, FDA, 2010. Accessed April 27, 2023: https://www.fda.gov/vaccines-blood-biologics/cellular-gene-therapy-products/provenge-sipuleucel-t
- Higano CS, Schellhammer PF, Small EJ, Burch PA, Nemunaitis J, Yuh L, Provost N, Frohlich MW. Integrated data from 2 randomized, double-blind, placebo-controlled, phase 3 trials of active cellular immunotherapy with sipuleucel-T in advanced prostate cancer. Cancer 2009;115(16):3670-9. https://doi.org/10.1002/cncr.24429

Product: Laviv (Azficel-T) **Year approved:** 2011

Sponsor: Fibrocell Technologies

Description: Autologous fibroblasts from skin punch biopsy

Indication: Improvement of the appearance nasolabial fold wrinkles in adults

Clinical Trial Structure: Two-arm [Laviv compared to placebo (medium without fibroblasts)]

Efficacy Endpoint: Score improvements on the Evaluator Wrinkle Assessment Scale and the Subject Wrinkle Assessment Scale

MOA:

- *Package Insert:* **"**The mechanism by which LAVIV improves the appearance of nasolabial fold wrinkles is unknown."
- *FDA SBRA:* "The mechanism of action of azficel-T has not been demonstrated. However, each lot is tested to determine that the product consists of viable fibroblasts that produce collagen. The potency of azficel-T is determined by the combination of cell count, viability, identity as fibroblasts and collagen content. The rationale for the choice of these characteristics is based on the premise that fibroblast survival and collagen biosynthesis following injection of azficel-T are likely to be important factors for the improvement in appearance of nasolabial fold wrinkles."
- *Smith et al, 2012:* "The exact mechanism of action of injected autologous fibroblasts remains unknown. They may exert their effect through one of several mechanisms. These could include the direct secretion of increased amounts of collagen and elastin, the induced proliferation of native fibroblasts, the secretion of cofactors that otherwise augment the dermal milieu, or simply multiplication of the transplanted fibroblasts. Most likely is it a combination of several of these processes."

Potency Testing (FDA SBRA): i) Cell count; ii) cell viability; iii) collagen production by the cells

References

- Summary Basis for Regulatory Action - Laviv, FDA, 2011. Accessed April 27, 2023: https://www.fda.gov/vaccines-blood-biologics/cellular-gene-therapy-products/laviv-azficel-t
- Package Insert - Laviv, FDA, 2011. Accessed April 27, 2023: https://www.fda.gov/vaccines-blood-biologics/cellular-gene-therapy-products/laviv-azficel-t
- Smith SR, Munavalli G, Weiss R, Maslowski JM, Hennegan KP, Novak JM. A multicenter, double-blind, placebo-controlled trial of autologous fibroblast therapy for the treatment of nasolabial fold wrinkles. Dermatol Surg 2012;38(7):1234-43. https://doi.org/10.1111/j.1524-4725.2012.02349.x

Product: Gintuit **Year approved:** 2012

Sponsor: Organogenesis

Description: Allogeneic cultured keratinocytes and fibroblasts in bovine collagen

Indication: For topical application to a surgically created vascular wound bed in the treatment of adult mucogingival conditions (not intended to provide root coverage)

Clinical Trial Structure: Two arm [Gintuit compared to standard of care in the same patient (gingival graft taken from the subject's palate)]

Efficacy Endpoints: Ability to regenerate ≥2 mm of keratinized gingiva at 6 months

MOA:

- *Package Insert:*
    - "Gintuit does not function as a tissue graft. The mechanism of action by which Gintuit increases keratinized tissue at the treated site has not been identified."
    - "The active ingredients of GINTUIT are the allogeneic keratinocytes, allogeneic dermal fibroblasts, and bovine Type I collagen. In vitro studies have shown that GINTUIT secretes human growth factors and cytokines, and contains extracellular matrix proteins. Growth factors, cytokines, and extracellular matrix proteins are known to be involved in wound repair and regeneration."
- *FDA Briefing Document, BLA 125400, November 17, 2011:* "The Committee noted that the histology assay is a good measure of the structural integrity of the product, however, the assay is not an adequate, sensitive measure of biological activity. While the exact biological metric that is most appropriate for product potency remains unclear, the Committee discussed that it would be appropriate to include cytokine assays given the current understanding of product function."

Potency Testing (FDA SBRA): Histology (hematoxylin and eosin staining) with morphological assessments: epidermal coverage, epidermal development, basal cell layer keratinocyte viability, suprabasal cell layer keratinocyte viability, dermal thickness, fibroblast density, and matrix integrity

References

- Summary Basis for Regulatory Action - Gintuit, FDA, 2012. Accessed April 27, 2023: https://www.fda.gov/vaccines-blood-biologics/cellular-gene-therapy-products/gintuit-allogeneic-cultured-keratinocytes-and-fibroblasts-bovine-collagen
- Package Insert – Gintuit, 2012. Accessed April 27, 2023: https://www.fda.gov/vaccines-blood-biologics/cellular-gene-therapy-products/gintuit-allogeneic-cultured-keratinocytes-and-fibroblasts-bovine-collagen
- FDA Briefing Document, BLA 125400. Cellular, Tissue and Gene Therapies Advisory Committee (CTGTAC) Meeting #54, FDA, November 17, 2011. Accessed May 2, 2023: https://www.fda.gov/vaccines-blood-biologics/cellular-gene-therapy-products/gintuit-questions-and-answers
- McGuire MK, Scheyer ET, Nevins ML, Neiva R, Cochran DL, Mellonig JT, Giannobile WV, Bates D. Living cellular construct for increasing the width of keratinized gingiva: results from a randomized, within-patient, controlled trial. J Periodontol 2011;82(10):1414-23. https://doi.org/10.1902/jop.2011.100671

Product: MACI (matrix-induced autologous chondrocyte implantation) **Year approved:** 2016

Sponsor: Vericel

Description: Autologous cultured chondrocytes on porcine collagen membrane

Indication: Full-thickness cartilage defects of the knee

Clinical Trial Structure: Two arm [MACI compared to standard of care (microfracture)]

Efficacy Endpoints: Knee injury and Osteoarthritis Outcome Score (KOOS) at 2 yrs

MOA (Package Insert): "No clinical pharmacology studies have been conducted with MACI and a mechanism of action has not been established."

Potency Testing:

- *FDA SBRA: i)* Cell number; ii) redacted; iii) redacted
- *Rapko et al., 2007:* PCR measurement of aggrecan gene expression

References

- Summary Basis for Regulatory Action - MACI, FDA, 2016. Accessed April 27, 2023: https://www.fda.gov/vaccines-blood-biologics/cellular-gene-therapy-products/maci-autologous-cultured-chondrocytes-porcine-collagen-membrane
- Package Insert – MACI, FDA, 2016. Accessed April 27, 2023: https://www.fda.gov/vaccines-blood-biologics/cellular-gene-therapy-products/maci-autologous-cultured-chondrocytes-porcine-collagen-membrane
- Rapko S, Parker A, Mortelliti C, Duguay SJ. #P192 - Aggrecan gene expression as a potency marker for matrix-induced autologous chondrocyte implantation (MACI). Osteoarthritis and Cartilage 15, Supplement B, page B136, 2007. https://doi.org/10.1016/S1063-4584(07)61547
- Saris D, Price A, Widuchowski W, Bertrand-Marchand M, Caron J, Drogset JO, Emans P, Podskubka A, Tsuchida A, Kili S, Levine D, Brittberg M. SUMMIT Study Group. Matrix-applied characterized autologous cultured chondrocytes versus microfracture: two-year follow-up of a prospective randomized trial. Am J Sports Med 2014, 42:1384-94. https://doi.org/10.1177/0363546514528093

Product: Stratagraft **Year approved:** 2021

Sponsor: Stratatech

Description: Allogeneic cultured keratinocytes and dermal fibroblasts in murine collagen

Indication: Deep partial-thickness thermal burns

Clinical Trial Structure: Two arm (Stratagraft compared to standard of care (autograft) in comparable wound sites of the same patient)

Efficacy Endpoints: i) Difference in the percent area of the treatments that required autografting by 3 months; ii) durable wound closure

MOA (Package Insert): "In vitro studies have shown that Sratagraft secretes human growth factors and cytokines and contains human ECM proteins. Growth factors, cytokines, and ECM are known to be involved in wound repair and regeneration."

Potency Testing (FDA SBRA): Redacted

References

- Summary Basis for Regulatory Action - Stratagraft, FDA, 2021. Accessed April 27, 2023: https://www.fda.gov/vaccines-blood-biologics/stratagraft
- Package Insert – Stratagraft, FDA, 2021. Accessed April 27, 2023: https://www.fda.gov/vaccines-blood-biologics/stratagraft
- Gibson ALF, Holmes IV JH, Shupp JW, Smith D, Joe V, Carson J, Litt J, Kahn S, Short T, Cancio L, Rizzo J, Carter JE, Foster K, Lokuta MA, Comer A, Smiell JM, Allen-Hoffmann BL. A phase 3, open-label, controlled, randomized, multicenter trial evaluating the efficacy and safety of StrataGraft1 construct in patients with deep partial-thickness thermal burns. Burns 2021;47:1024-1037. https://doi.org/10.1016/j.burns.2021.04.021

Product: Rethymic **Year approved:** 2021

Sponsor: Enzyvant Therapeutics

Description: Allogeneic processed thymus tissue

Indication: Congenital athymia

Clinical Trial Structure: Single arm (compared to historical data for standard of care)

Efficacy Endpoint: Survival

MOA:

- *Package Insert:* "Rethymic is intended to reconstitute immunity in patients who are athymic. The proposed mechanism of action involves the migration of recipient T-cell progenitors from the bone marrow to the implanted Rethymic slices, where they develop into naïve immunocompetent recipient T-cells. Evidence of thymic function can be observed with the development of naïve T-cells in the peripheral blood."
- *FDA SBRA:*
    - "Rethymic is intended to function as if it is a normal endogenous thymus. Its thymic endothelial cells recruit immature host T-cells (thymocytes) into the slices where they undergo further maturation and positive and negative selection, releasing into circulation immunocompetent naive T-cells that are capable of providing protection from infection."
    - **"**The proposed mechanism of action is migration of the recipient's bone marrow-derived T-cell progenitors into the thymic allograft where they are "educated" to produce immunocompetent T-cells that are tolerant of both donor and recipient tissues while maintaining the ability to respond to foreign antigens."

Potency Testing (FDA SBRA): Histology-based (for tissue organization, viability and retention of important cell types believed to be important for product function)

Potency Comments (FDA SBRA): "Histological evaluation, including for potency, by histology is reasonable for a tissue-based product, though is limited by sensitivity and variabilities inherent in the method."

References

- Summary Basis for Regulatory Action - Rethymic, FDA, 2021. Accessed April 27, 2023: https://www.fda.gov/vaccines-blood-biologics/rethymic
- Package Insert – Rethymic, FDA, 2021. Accessed April 27, 2023: https://www.fda.gov/vaccines-blood-biologics/rethymic
- Markert ML, Gupton SE, McCarthy EA. Experience with cultured thymus tissue in 105 children. J Allergy Clin Immunol 2022;149:747-57. https://doi.org/10.1016/j.jaci.2021.06.028

Product: Zynteglo (betibeglogene autotemcel) **Year approved:** 2022

Sponsor: Bluebird Bio

Description: Zynteglo consists of an autologous CD34+ cell-enriched population that contains the patient's own hematopoietic stem cells (HSCs) transduced ex vivo with the BB305 lentiviral vector (LVV) encoding β^{A-T87Q}-globin

Indication: For treatment of adult and pediatric patients with ß-thalassemia who require regular red blood cell (RBC) transfusions

Clinical Trial Structure: Single arm (compared to patient symptoms prior to treatment)

Efficacy Endpoint: Transfusion independence (TI) defined as weighted average hemoglobin (Hb) ≥ 9 g/dL without RBC transfusions for ≥ 12 months at any time after Zynteglo infusion

MOA (Package Insert): "Zynteglo adds functional copies of a modified β-globin gene into patients' hematopoietic stem cells (HSCs) through transduction of autologous CD34+ cells with BB305 LVV. After Zynteglo infusion, transduced CD34+ HSCs engraft in the bone marrow and differentiate to produce RBCs containing biologically active β^{A-T87Q}-globin (a modified β-globin protein) that will combine with α-globin to produce functional adult Hb containing β^{A-T87Q}-globin (HbAT87Q). β^{A-T87Q}-globin can be quantified relative to other globin species in peripheral blood using high-performance liquid chromatography. β^{A-T87Q}-globin expression is designed to correct the β/α-globin imbalance in erythroid cells of patients with β-thalassemia and has the potential to increase functional adult HbA and total Hb to normal levels and eliminate dependence on regular pRBC transfusions."

Potency Testing (FDA SBRA): i) Vector copy number (qPCR); ii) percent LVV+ cells; iii) colony forming cells; iv) β^{A-T87Q}-globin quantitative protein expression; v) redacted; vi) redacted

References

- Summary Basis for Regulatory Action - Zynteglo, FDA, 2022. Accessed April 26, 2023: https://www.fda.gov/vaccines-blood-biologics/zynteglo
- Package Insert - Zynteglo, FDA, 2022. Accessed April 26, 2023: https://www.fda.gov/vaccines-blood-biologics/zynteglo
- Locatelli F, Thompson AA, Kwiatkowski JL, Porter JB, Thrasher AJ, Hongeng S, Sauer MG, Thuret I, Lal A, Algeri M, Schneiderman J, Olson TS, Carpenter B, Amrolia PJ, Anurathapan U, Schambach A, Chabannon C, Schmidt M, Labik I, Elliot H, Guo R, Asmal M, Colvin RA, Walters MC. Betibeglogene Autotemcel Gene Therapy for Non-β^0/β^0 Genotype β-Thalassemia. N Engl J Med 2022;386(5):415-427. https://doi.org/10.1056/NEJMoa2113206

Product: Skysona (elivaldogene autotemcel) **Year approved:** 2022

Sponsor: Bluebird Bio

Description: Skysona consists of an autologous CD34+ cell-enriched population, that contains the patient's own hematopoietic stem cells (HSCs), transduced ex vivo with the Lenti-D lentiviral vector (LVV) containing the ATP-binding cassette, sub-family D, member 1 (ABCD1) gene encoding the adrenoleukodystrophy protein (ALDP).

Indication: To slow the progression of neurologic dysfunction in boys 4-17 years of age with early, active cerebral adrenoleukodystrophy (CALD)

Clinical Trial Structure: Single arm (compared to historical data)

Efficacy Endpoint: Time from onset of symptoms to first major functional disability (MFD) or death (MFD defined as loss of communication, cortical blindness, requirement for tube feeding, total incontinence, wheelchair dependence, or complete loss of voluntary movement)

MOA (Package Insert): "Skysona adds functional copies of the ABCD1 cDNA into patients' hematopoietic stem cells (HSCs) through transduction of autologous CD34+ cells with Lenti-D LVV. After Skysona infusion, transduced CD34+ HSCs engraft in the bone marrow and differentiate into various cell types, including monocytes (CD14+) capable of producing functional ALDP. Functional ALDP can then participate in the local degradation of very long chain fatty acids (VLCFAs), which is believed to slow or possibly prevent further inflammation and demyelination."

Potency Testing (FDA SBRA): i) Vector copy number (qPCR); ii) percent LVV+ cells; iii) percent ADLP+ cells; iv) redacted; v) redacted; vi) redacted

References

- Summary Basis for Regulatory Action - Skysona, FDA, 2022. Accessed April 26, 2023: https://www.fda.gov/vaccines-blood-biologics/skysona
- Package Insert - Skysona, FDA, 2022. Accessed April 26, 2023: https://www.fda.gov/vaccines-blood-biologics/skysona

Product: Omisirge (omidubicel) **Year approved:** 2023

Sponsor: Gamida Cell Ltd.

Description: Omisirge is a nicotinamide modified allogeneic hematopoietic progenitor cell (HPC) therapy derived from cord blood. Omisirge contains two cell fractions from the same allogeneic cord blood unit (CBU): (1) ex vivo cultured fraction (CF) of CD34+ cells that will engraft, and (2) a supportive non-cultured fraction (NF) of the non-selected CBU cells. The CF is a yellowish suspension of selected hematopoietic CD34+ progenitor cells ex vivo cultured with nicotinamide (NAM). In addition to the CD34+ progenitor cells, the CF consists of other cell populations, including lineage committed myelomonocytic cells, dendritic cells and granulocytes. The NF is a reddish suspension consisting of allogeneic, hematopoietic mature myeloid and lymphoid cells collected from the non-selected cells.

Indication: For use in adults and pediatric patients 12 years and older with hematologic malignancies who are planned for umbilical cord blood transplantation following myeloablative conditioning to reduce the time to neutrophil recovery and the incidence of infection

Clinical Trial Structure: Two-arm (compared to umbilical cord blood transplantation)

Efficacy Endpoint: i) time to neutrophil recovery following transplantation and ii) the incidence of Grade 2/3 bacterial or Grade 3 fungal infections through Day 100 following transplantation

MOA (Package Insert): "Omisirge is a nicotinamide (NAM) modified allogeneic hematopoietic progenitor cell therapy derived from cord blood used as an allogeneic stem cell donor source. Omisirge is manufactured utilizing a proprietary NAM based technology producing enriched HPCs. NAM technology overcomes the induction of accelerated proliferation, differentiation, cellular stress and signaling pathways that are typically activated when HPCs are removed from their natural environment. Ex-vivo culturing of cord blood derived HPCs in the presence of NAM leads to preservation of their stemness, homing to the bone marrow (BM) and retained engraftment capacity as demonstrated by rapid neutrophil engraftment and multi lineage immune reconstitution as observed in the clinical trials with Omisirge."

Potency Testing (FDA SBRA): CD34+ cell fold-increase

References

- Package Insert - Omisirge, FDA, 2023. Accessed April 26, 2023: https://www.fda.gov/vaccines-blood-biologics/omisirge
- Summary Basis for Regulatory Action - Omisirge, FDA, 2023. Accessed May 24, 2023: https://www.fda.gov/vaccines-blood-biologics/omisirge
- Lin C, Schwarzbach A, Sanz J, Montesinos P, Stiff P, Parikh S, Brunstein C, Cutler C, Lindemans CA, Hanna R, Koh LP, Jagasia MH, Valcarcel D, Maziarz RT, Keating AK, Hwang WYK, Rezvani AR, Karras NA, Fernandes JF, Rocha V, Badell I, Ram R, Schiller GJ, Volodin L, Walters MC, Hamerschlak N, Cilloni D, Frankfurt O, McGuirk JP, Kurtzberg J, Sanz G, Simantov R, Horwitz ME. Multicenter Long-Term Follow-Up of Allogeneic Hematopoietic Cell Transplantation with Omidubicel: A Pooled Analysis of Five Prospective Clinical Trials. Transplant Cell Ther 2023;29(5):338.e1-338.e6. https://doi.org/10.1016/j.jtct.2023.01.031

Product: Lantidra (donislecel-jujn) **Year approved:** 2023

Sponsor: CellTrans Inc.

Description: Allogeneic pancreatic islet cellular therapy. LANTIDRA is the first marketed cell-based therapy made from deceased allogeneic donor pancreatic islets of Langerhans (cluster of cells within the pancreas) for the treatment of T1D in adults who are unable to approach target HbA1c because of current repeated episodes of severe hypoglycemia despite intensive diabetes management and education. LANTIDRA is composed of mixed populations of endocrine cells, including beta cells that produce insulin. At least 30% of the product is made of insulin-producing beta cells. Together, the cells composing LANTIDRA regulate blood glucose levels through secretion of hormones in response to glucose stimulation. LANTIDRA is a suspension of islet cells administered through the hepatic portal vein.

Indication: The treatment of adults with Type 1 diabetes who are unable to approach target HbA1c because of current repeated episodes of severe hypoglycemia despite intensive diabetes management and education.

Clinical Trial Structure: Single arm

Efficacy Endpoint: Insulin independence

MOA (Package Insert): "Pancreatic islets regulate blood glucose levels through secretion of multiple hormones in response to increases and decreases in blood glucose. Endocrine cells within pancreatic islets release insulin, glucagon, somatostatin, pancreatic peptide, and ghrelin. Insulin stimulates glucose uptake by peripheral tissues; glucagon mobilizes glucose from the liver into circulation; somatostatin inhibits both α- and β-cell secretions; pancreatic peptide inhibits pancreatic exocrine secretion; and ghrelin inhibits insulin secretion. The primary mechanism of action of LANTIDRA is believed to be secretion of insulin by infused(transplanted) β-cells."

Potency Testing (FDA Advisory Committee Meeting): i) Glucose Stimulation Index (GSI): ELISA (enzyme linked immunosorbent assay) quantification of insulin release in glucose stimulated islets; ii) Islet Yield: Dithizone (DTZ) stain and microscopic quantification; iii) Viability: SYTO 13 green/ethidium bromide staining and microscopic evaluation

References

- Package Insert - Lantidra, FDA, 2023. Accessed July 14, 2023: https://www.fda.gov/vaccines-blood-biologics/lantidra
- Summary Basis for Regulatory Action - Lantidra, FDA, 2023. Accessed August 9, 2023: https://www.fda.gov/vaccines-blood-biologics/lantidra
- FDA Cellular, Tissue and Gene Therapies Advisory Committee Meeting, AM Session Product Characterization - BLA 125734 (Lantidral; donislecel), April 15, 2021. Accessed July 17, 2023: https://www.fda.gov/advisory-committees/advisory-committee-calendar/cellular-tissue-and-gene-therapies-advisory-committee-april-15-2021-meeting-announcement-04152021
- Qi M, Kinzer K, Danielson KK, Martellotto J, Barbaro B, Wang Y, Bui JT, Gaba RC, Knuttinen G, Garcia-Roca R, Tzvetanov I, Heitman A, Davis M, McGarrigle JJ, Benedetti E, Oberholzer J. Five-year follow-up of patients with type 1 diabetes transplanted with allogeneic islets: the UIC experience. Acta Diabetol 2014;51(5):833-43. https://doi.org/10.1007/s00592-014-0627-6

Product: Casgevy (exagamglogene autotemcel) **Year approved:** 2023

Sponsor: Vertex Pharmaceuticals, Inc.

Description (Package Insert): "CASGEVY is a cellular gene therapy consisting of autologous CD34+ HSCs edited by CRISPR/Cas9-technology at the erythroid specific enhancer region of the BCL11A gene to reduce BCL11A expression in erythroid lineage cells, leading to increased fetal hemoglobin (HbF) protein production. CASGEVY is prepared from the patient's own HSCs, which are obtained via apheresis procedure(s). The autologous cells are enriched for CD34+ cells, and then genome edited ex vivo by introducing the CRISPR/Cas9 ribonucleoprotein (RNP) complex by electroporation. The guide RNA included in the RNP complex enables CRISPR/Cas9 to make a precise DNA double-strand break at a critical transcription factor binding site (GATA1) in the erythroid specific enhancer region of the BCL11A gene. As a result of the editing, GATA1 binding is disrupted and BCL11A expression is reduced. This reduction in BCL11A expression conversely results in an increase in gamma-globin expression and downstream fetal hemoglobin formation." "CASGEVY is provided as a single dose for infusion containing a suspension of CD34+ cells in one or more vials." "The minimum recommended dose of CASGEVY is 3×10^6 CD34+ cells/kg."

Indication: Treatment of patients aged 12 years and older with transfusion-dependent ß-thalassemia (TDT).

Clinical Trial Structure: Single arm

Efficacy Endpoint: The proportion of patients who did not experience any protocol-defined severe vaso-occlusive crises (VOCs) for at least 12 consecutive months within the first 24 months after CASGEVY infusion. VOCs were defined as: i) acute pain event requiring a visit to a medical facility and administration of pain medications (opioids or intravenous [IV] non-steroidal anti-inflammatory drugs [NSAIDs]) or RBC transfusions; ii) acute chest syndrome; iii) priapism lasting > 2 hours and requiring a visit to a medical facility; and iv) splenic sequestration.

MOA (Package Insert): "After CASGEVY infusion, the edited CD34+ cells engraft in the bone marrow and differentiate to erythroid lineage cells with reduced BCL11A expression. Reduced BCL11A expression results in an increase in γ-globin expression and HbF protein production in erythroid cells. In patients with severe sickle cell disease, HbF expression reduces intracellular hemoglobin S (HbS) concentration, preventing the red blood cells from sickling and addressing the underlying cause of disease, thereby eliminating VOCs."

Potency Testing (SBRA): i) On-target editing frequency (tracking of indels by decomposition, TIDE); ii) redacted; iii) redacted.

References

- Package Insert - Casgevy, FDA, 2023. Accessed February 14, 2024: https://www.fda.gov/vaccines-blood-biologics/casgevy
- Summary Basis for Regulatory Action - Casgevy, FDA, 2023. Accessed February 14, 2024: https://www.fda.gov/vaccines-blood-biologics/casgevy
- Frangoul H, Altshuler D, Cappellini MD, Chen YS, Domm J, Eustace BK, Foell J, de la Fuente J, Grupp S, Handgretinger R, Ho TW, Kattamis A, Kernytsky A, Lekstrom-Himes J, Li AM, Locatelli F, Mapara MY, de Montalembert M, Rondelli D, Sharma A, Sheth S, Soni S, Steinberg MH, Wall D, Yen A, Corbacioglu S. CRISPR-Cas9 Gene Editing for Sickle Cell Disease and β-Thalassemia. N Engl J Med. 2021 Jan 21;384(3):252-260. https://doi.org/10.1056/NEJMoa2031054

Product: Lyfgenia (lovotibeglogene autotemcel) **Year approved:** 2023

Sponsor: Bluebird Bio

Description (Package Insert): "Lygenia is a $\beta^{A\text{-}T87Q}$-globin gene therapy consisting of autologous CD34+ cells from patients with sickle cell disease containing hematopoietic stem cells (HSCs) transduced with BB305 LVV encoding $\beta^{A\text{-}T87Q}$-globin, suspended in cryopreservation solution. LYFGENIA is intended for one-time administration to add functional copies of a modified form of the β-globin gene ($\beta^{A\text{-}T87Q}$-globin gene) into the patient's own HSCs. LYFGENIA is prepared using the patient's own HSCs, which are collected via apheresis procedure(s). The autologous cells are enriched for CD34+ cells, then transduced ex vivo with BB305 LVV. The promoter, a regulatory element that controls the expression of the transgene selected for BB305 LVV, is a cellular (non-viral) promoter that controls gene expression specific to the erythroid lineage cells (red blood cells and their precursors). BB305 LVV encodes $\beta^{A\text{-}T87Q}$-globin."

Indication: Treatment of patients 12 years of age or older with sickle cell disease and a history of vaso-occlusive events (VOEs).

Clinical Trial Structure: Single arm

Efficacy Endpoint: Complete resolution of VOEs and severe VOEs between 6 months and 18 months after infusion of LYFGENIA. VOEs were defined as any of the following events requiring evaluation at a medical facility: i) an episode of acute pain with no medically determined cause other than vaso-occlusion, lasting more than 2 hours; ii) acute chest syndrome (ACS); iii) acute hepatic sequestration; or iv) acute splenic sequestration. Severe VOE (sVOE) were defined as either of the following events: i) VOE requiring a hospitalization or multiple visits to an emergency department/urgent care over 72 hours and receiving intravenous medications at each visit; or ii) priapism requiring any level of medical attention.

MOA (Package Insert): "LYFGENIA adds functional copies of a modified βA-globin gene (threonine [T] replaced with glutamine [Q] at position 87, T87Q or $\beta^{A\text{-}T87Q}$-globin) into patients' hematopoietic stem cells (HSCs) through transduction of autologous CD34+ cells with BB305 LVV. After LYFGENIA infusion, the transduced CD34+ HSCs engraft in the bone marrow and differentiate to produce red blood cells containing biologically active $\beta^{A\text{-}T87Q}$-globin that will combine with α-globin to produce functional Hb containing $\beta^{A\text{-}T87Q}$-globin (HbA^{T87Q}). $\beta^{A\text{-}T87Q}$-globin can be distinguished from wildtype β^{A}-globin and from β^{S}-globin through reverse-phase high-performance liquid chromatography (RPHPLC) or ultra-high performance liquid chromatography (UPLC). HbA^{T87Q} has similar oxygen-binding affinity and oxygen hemoglobin dissociation curve to wild type HbA, reduces intracellular and total hemoglobin S (HbS) levels, and is designed to sterically inhibit polymerization of HbS thereby limiting the sickling of red blood cells."

Potency Testing (SBRA): i) Vector copy number; ii) redacted, iii) redacted; iv) redacted; v) redacted; vi) $\beta^{A\text{-}T87Q}$-globin quantitative protein expression.

References

- Package Insert - Lyfgenia, FDA, 2023. Accessed ed February 14, 2024: https://www.fda.gov/vaccines-blood-biologics/lyfgenia
- Summary Basis for Regulatory Action - Lyfgenia, FDA, 2023. Accessed February 14, 2024: https://www.fda.gov/vaccines-blood-biologics/lyfgenia
- Kanter J, Walters MC, Krishnamurti L, Mapara MY, Kwiatkowski JL, Rifkin-Zenenberg S, Aygun B, Kasow KA, Pierciey FJ Jr, Bonner M, Miller A, Zhang X, Lynch J, Kim D, Ribeil JA, Asmal M, Goyal S, Thompson AA, Tisdale JF. Biologic and Clinical Efficacy of LentiGlobin for Sickle Cell Disease. N Engl J Med. 2022 Feb 17;386(7):617-628. https://doi.org/10.1056/NEJMoa2117175

Product: Amtagvi (lifileucel) **Year approved:** 2024

Sponsor: Iovance Biotherapeutics, Inc.

Description (Package Insert): AMTAGVI (lifileucel) is a tumor-derived autologous T cell immunotherapy comprised of a suspension of tumor-derived T cells for intravenous infusion. AMTAGVI is manufactured from resected patient tumor tissue prosected from one or more tumor lesions. Immune cells derived from a patient's tumor(s) are expanded in cell culture, washed, formulated as a cell suspension, and cryopreserved. The product must pass a sterility test before release for shipping as a frozen suspension in 1 to 4 patient-specific infusion bag(s) in individual protective metal cassettes. The product is thawed prior to administration back into the same patient. AMTAGVI is composed primarily of T cells of the CD4+T and CD8+T cell lineages. AMTAGVI may also contain monocytes and other lymphocytes, including B cells and NK cells. AMTAGVI may contain viable melanoma tumor cells from the original tumor tissue used to manufacture the product.

Indication: Indicated for the treatment of adult patients with unresectable or metastatic melanoma previously treated with a PD-1 blocking antibody, and if BRAF V600 mutation positive, a BRAF inhibitor with or without a MEK inhibitor.

Clinical Trial Structure: Single arm

Efficacy Endpoint: Objective response rate according to the RECIST guidelines (Response Evaluation Criteria In Solid Tumors), which is the fraction of patients demonstrating a complete response or partial response. Response is based on tumor load, which is assessed by measuring the longest axis of lesions identified during a baseline examination. The size of lesions can be measured by various means depending on the location: physical examination, chest x-ray, computed tomography, magnetic resonance imaging, ultrasound, etc. The RECIST criteria are as follows:

- *Complete response*—the disappearance of all target lesions;
- *Partial response*—at least a 30% decrease in the sum of the longest diameter of target lesions
- *Progressive disease*—at least a 20% increase in the sum of the longest diameter of target lesions
- *Stable disease*—neither sufficient shrinkage to qualify for partial response nor sufficient increase to qualify for progressive disease

MOA (Package Insert): "The specific mechanism of action of AMTAGVI (lifileucel) is unknown."

Potency Testing (SBRA): i) redacted, ii) redacted, iii) redacted, iv) redacted, v) dose (total viable cells), vi) redacted and vii) redacted.

References

- Package Insert - Amtagvi, FDA, 2024. Accessed ed February 20, 2024: https://www.fda.gov/vaccines-blood-biologics/amtagvi
- Summary Basis for Regulatory Action - Amtagvi, FDA, 2024. Accessed March 11, 2024: https://www.fda.gov/vaccines-blood-biologics/amtagvi
- Sarnaik AA, Hamid O, Khushalani NI, Lewis KD, Medina T, Kluger HM, Thomas SS, Domingo-Musibay E, Pavlick AC, Whitman ED, Martin-Algarra S, Corrie P, Curti BD, Oláh J, Lutzky J, Weber JS, Larkin JMG, Shi W, Takamura T, Jagasia M, Qin H, Wu X, Chartier C, Graf Finckenstein F, Fardis M, Kirkwood JM, Chesney JA. Lifileucel, a Tumor-Infiltrating Lymphocyte Therapy, in Metastatic Melanoma. J Clin Oncol. 2021;39(24):2656-2666. https://doi.org/10.1200/JCO.21.00612
- Chesney J, Lewis KD, Kluger H, Hamid O, Whitman E, Thomas S, Wermke M, Cusnir M, Domingo-Musibay E, Phan GQ, Kirkwood JM, Hassel JC, Orloff M, Larkin J, Weber J, Furness AJS, Khushalani NI, Medina T, Egger ME, Graf Finckenstein F, Jagasia M, Hari P, Sulur G, Shi W, Wu X, Sarnaik A. Efficacy and safety of lifileucel, a one-time autologous tumor-infiltrating lymphocyte (TIL) cell therapy, in patients with advanced melanoma after progression on immune checkpoint inhibitors and targeted therapies: pooled analysis of consecutive cohorts of the C-144-01 study. J Immunother Cancer. 2022;10(12):e005755. https://doi.org/10.1136/jitc-2022-005755
- Therasse P, Arbuck SG, Eisenhauer EA, Wanders J, Kaplan RS, Rubinstein L, Verweij J, Van Glabbeke M, van Oosterom AT, Christian MC, Gwyther SG. New guidelines to evaluate the response to treatment in solid tumors. European Organization for Research and Treatment of Cancer, National Cancer Institute of the United States, National Cancer Institute of Canada. J Natl Cancer Inst. 2000 Feb 2;92(3):205-16. https://doi.org/10.1093/jnci/92.3.205
