# Supplementary material for: Mechanism of action, potency and efficacy: considerations for cell therapies
Source: J Transl Med. 2024 May 2;22:416. doi: 10.1186/s12967-024-05179-7 (PMC11067168; doi:10.1186/s12967-024-05179-7)
Supplement: Supplementary file 2 — Additional file 2. Key definitions that are referred to in the main text. [file 12967_2024_5179_MOESM2_ESM.pdf]

Supplementary File 2: Definitions Cited in the Manuscript

Simon et al., Mechanism of Action, Potency and Efficacy: Considerations for Cell Therapies

Measurand: “the quantity or property intended to be measured”

> *Source:* International Vocabulary of Metrology - Basic and General Concepts and Associated Terms (VIM), 3rd ed.; Joint Committee for Guides in Metrology (JCGM), 2012. Accessed June 1, 2023: https://www.bipm.org/utils/common/documents/jcgm/JCGM_200_2012.pdf

Measurement: “process of experimentally obtaining one or more quantity values that can reasonably be attributed to a quantity”

> *Source:* International Vocabulary of Metrology - Basic and General Concepts and Associated Terms (VIM), 3rd ed.; Joint Committee for Guides in Metrology (JCGM), 2012. Accessed June 1, 2023: https://www.bipm.org/utils/common/documents/jcgm/JCGM_200_2012.pdf

Mechanism of action (MOA): “pharmacologic action at the receptor, membrane, or tissue level”

> *Source:* Guidance for Industry and Review Staff - Labeling for Human Prescription Drug and Biological Products — Determining Established Pharmacologic Class for Use in the Highlights of Prescribing Information - Good Review Practice. US Food and Drug Administration, 2009. Accessed June 1, 2023: https://www.fda.gov/media/77834/download

Mode of action: “the means by which a product achieves its intended therapeutic effect or action”

> *Source:* 21 CFR 3.2(k), Code of Federal Regulations. US National Archives. Accessed June 1, 2023: https://www.ecfr.gov/current/title-21/chapter-I/subchapter-A/part-3/subpart-A/section-3.2

Potency: “The specific ability or capacity of the product, as demonstrated by appropriate laboratory tests or by adequately controlled clinical data obtained through the administration of the product in the manner intended, to effect a given result.”

> *Source:* 21 CFR 600.3(s), Code of Federal Regulations, US National Archives. Accessed June 1, 2023: https://www.ecfr.gov/current/title-21/chapter-I/subchapter-F/part-600/subpart-A/section-600.3#p-600.3(s)

Potency: “The measure of the biological activity using a suitably quantitative biological assay (also called potency assay or bioassay), based on the attribute of the product which is linked to the relevant biological properties.”

> *Source:* International Conference on Harmonisation (ICH). ICH Harmonised Tripartite Guideline Specifications: Test Procedures And Acceptance Criteria For Biotechnological/Biological Products Q6B (ICH Q6B), Current Step 4 version, 1999. Accessed May 8, 2023: https://www.ich.org/page/quality-guidelines

Potency: "measure of the biological activity using a suitably quantitative analytical method, based on the attribute of the product which is linked to the relevant biological properties"

> *Source:* ISO 23033 - Biotechnology — Analytical methods — General requirements and considerations for the testing and characterization of cellular therapeutic products. International Organization for Standardization: Geneva, Switzerland, 2021.

Potency test: Tests for potency shall consist of either in vitro or in vivo tests, or both, which have been specifically designed for each product so as to indicate its potency in a manner adequate to satisfy the interpretation of potency given by the definition in § 600.3(s) of this chapter.

> *Source:* 21 CFR 610.10, Code of Federal Regulations, US National Archives. Accessed February 15, 2024: https://www.ecfr.gov/current/title-21/chapter-I/subchapter-F/part-610/subpart-B/section-610.10

Efficacy: "Evidence consisting of adequate and well-controlled investigations, including clinical investigations, by experts qualified by scientific training and experience to evaluate the effectiveness of the drug involved, on the basis of which it could fairly and responsibly be concluded by such experts that the drug will have the effect it purports or is represented to have under the conditions of use prescribed, recommended, or suggested in the labeling or proposed labeling thereof."

> *Source:* Guidance for Industry - Providing Clinical Evidence of Effectiveness for Human Drug and Biological Products. Food and Drug Administration, 1998. Accessed May 31, 2023: https://www.fda.gov/files/drugs/published/Providing-Clinical-Evidence-of-Effectiveness-for-Human-Drug-and-Biological-Products..pdf

Efficacy endpoint: "Measures intended to reflect the effects of a drug. They include assessments of clinical events (e.g., mortality, stroke, pulmonary exacerbation, venous thromboembolism), patient symptoms (e.g., pain, dyspnea, depression), measures of function (e.g., ability to walk or exercise), or surrogates of these events or symptoms."

> *Source:* Draft Guidance for Industry - Multiple Endpoints in Clinical Trials, US Food and Drug Administration, 2017. Accessed May 25, 2023: https://www.fda.gov/files/drugs/published/Multiple-Endpoints-in-Clinical-Trials-Guidance-for-Industry.pdf
>
> *Note: The term "efficacy endpoint" is used interchangeably with "clinical outcome".*

Clinical outcome: "Clinical outcomes directly measure whether people in a trial feel or function better or live longer."

> *Source:* Surrogate Endpoint Resources for Drug and Biologic Development, US Food and Drug Administration. Accessed May 25, 2023: https://www.fda.gov/drugs/development-resources/surrogate-endpoint-resources-drug-and-biologic-development
>
> *Note: The phrase "feels, functions or survives" is often used.*
